# Proteomics informed by transcriptomics for characterising active transposable elements and genome annotation in *Aedes aegypti*

**DOI:** 10.1186/s12864-016-3432-5

**Published:** 2017-01-19

**Authors:** Kevin Maringer, Amjad Yousuf, Kate J. Heesom, Jun Fan, David Lee, Ana Fernandez-Sesma, Conrad Bessant, David A. Matthews, Andrew D. Davidson

**Affiliations:** 1 School of Cellular and Molecular Medicine, University of Bristol, Bristol, BS8 1TD UK; 2 Department of Microbiology, Icahn School of Medicine at Mount Sinai, New York, 10029 NY USA; 3 College of Applied Medical Sciences, Taibah University, Medina, Kingdom of Saudi Arabia; 4 School of Biochemistry, University of Bristol, Bristol, BS8 1TD UK; 5 School of Biological and Chemical Sciences, Queen Mary University of London, London, E1 4NS UK; 6 Present address: Department of Microbial Sciences, University of Surrey, Guildford, GU2 7XH UK

**Keywords:** *Aedes aegypti*, Genome annotation, Non-model organism, PIT, Proteomics informed by transcriptomics, Transposon

## Abstract

**Background:**

*Aedes aegypti* is a vector for the (re-)emerging human pathogens dengue, chikungunya, yellow fever and Zika viruses. Almost half of the *Ae. aegypti* genome is comprised of transposable elements (TEs). Transposons have been linked to diverse cellular processes, including the establishment of viral persistence in insects, an essential step in the transmission of vector-borne viruses. However, up until now it has not been possible to study the overall proteome derived from an organism’s mobile genetic elements, partly due to the highly divergent nature of TEs. Furthermore, as for many non-model organisms, incomplete genome annotation has hampered proteomic studies on *Ae. aegypti*.

**Results:**

We analysed the *Ae. aegypti* proteome using our new proteomics informed by transcriptomics (PIT) technique, which bypasses the need for genome annotation by identifying proteins through matched transcriptomic (rather than genomic) data. Our data vastly increase the number of experimentally confirmed *Ae. aegypti* proteins. The PIT analysis also identified hotspots of incomplete genome annotation, and showed that poor sequence and assembly quality do not explain all annotation gaps. Finally, in a proof-of-principle study, we developed criteria for the characterisation of proteomically active TEs. Protein expression did not correlate with a TE’s genomic abundance at different levels of classification. Most notably, long terminal repeat (LTR) retrotransposons were markedly enriched compared to other elements. PIT was superior to ‘conventional’ proteomic approaches in both our transposon and genome annotation analyses.

**Conclusions:**

We present the first proteomic characterisation of an organism’s repertoire of mobile genetic elements, which will open new avenues of research into the function of transposon proteins in health and disease. Furthermore, our study provides a proof-of-concept that PIT can be used to evaluate a genome’s annotation to guide annotation efforts which has the potential to improve the efficiency of annotation projects in non-model organisms. PIT therefore represents a valuable new tool to study the biology of the important vector species *Ae. aegypti*, including its role in transmitting emerging viruses of global public health concern.

**Electronic supplementary material:**

The online version of this article (doi:10.1186/s12864-016-3432-5) contains supplementary material, which is available to authorized users.

## Background

**Fig. 1:**
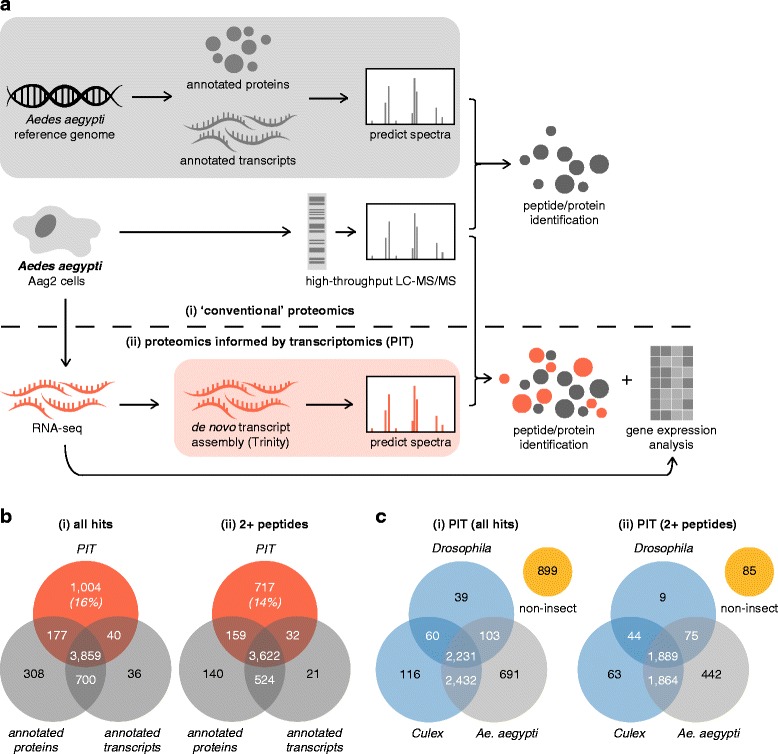
PIT identifies additional proteins in *Ae. aegypti* cells compared to ‘conventional’ proteomics. **a** Overview of the PIT pipeline. In ‘conventional’ proteomics (i), proteins detected by high-throughput LC-MS/MS from *Ae. aegypti* cell extracts are identified by comparison to mass spectra computationally predicted from protein or transcript annotations on the *Ae. aegypti* reference genome. (Annotated transcripts are *in silico* translated prior to mass spectra prediction). PIT identifies additional proteins by using RNA-seq to identify transcripts in RNA samples matched to protein isolates (ii). Transcripts are assembled *de novo* using Trinity software, translated *in silico*, and used for mass spectra prediction for peptide identification. From a single experimental sample, proteins are identified without the need for an annotated reference genome, and transcript abundance can be inferred from RNA-seq data. **b** Total unique proteins (i) and proteins with at least two recorded peptides (ii) identified in Aag2 cells based on the *Ae. aegypti* reference genome protein or transcript annotations, or using PIT. Percentages indicate the proportion of proteins identified only by PIT. **c** BLAST analysis of the PIT-identified proteome. Hits were mapped against the *Ae. aegypti* [taxid 7159], *Culex quinquefasciatus* [taxid 7176] (*Culex*) or *Drosophila melanogaster* [taxid 7227] (*Drosophila*) Ref-Seq databases. A subset of hits did not match annotated genes from these dipteran insects (non-insect). (i) Total PIT proteome, (ii) Translated ORFs from Trinity transcripts matched with at least two peptides

The arrival of the ‘omics’ era has revolutionised the study of model organisms, including humans and mice, and even greater gains are arguably being made in less tractable non-model organisms. The number of organisms with sequenced genomes is increasing rapidly, facilitating proteomic, transcriptomic and molecular studies. However, proteomic studies have been hampered in organisms with unsequenced or incompletely annotated genomes [1]. This is because proteomics usually relies on genome annotation for identifying peptides detected by high-throughput liquid chromatography with coupled tandem mass spectrometry (LC-MS/MS) (Fig. 1Ai). We previously reported a new approach called proteomics informed by transcriptomics (PIT) that circumvents the requirement for a reference genome by identifying peptides based on transcripts assembled *de novo* from RNA-seq data (Fig. 1Aii) [2]. Importantly, especially for non-model species, we showed that the approach was universal and comparable to using gold standard bioinformatic datasets in humans. Amongst other non-model organisms, PIT has been applied to reservoir hosts and arthropod vectors of infectious diseases, including bats and ticks [3–6]. While proteomic data can provide genome annotation [1, 7], whether PIT can evaluate the state of a genome’s annotation has not been tested. Here, we used the reference genome sequence for the important vector mosquito *Aedes aegypti* [8] to assess PIT’s utility in evaluating genome annotation. The *Ae. aegypti* genome is particularly amenable to such studies because it is in an intermediate state of annotation, less complete than the human genome, but more advanced than that of other non-model organisms.

We also investigated the usefulness of PIT to study the ‘mobilome’ (the total of all mobile transposable elements (TEs) in a genome) in *Ae. aegypti* cells. While several transposon classification systems have been proposed (for example [9–12]), we will here use conventions described by Tu et al. [10], because this system is specific to mosquitoes, and because it aligns with the major database used in our analyses (TEfam, tefam.biochem.vt.edu) and with TE classifications used by Nene et al. in the published *Ae. aegypti* reference genome [8]. As described by Tu et al., mosquito TEs can be divided into two major classes based on their mechanism of transposition. Class I TEs replicate via a reverse transcriptase-generated RNA intermediate and result in amplification of the element, while class II transposons transpose without RNA intermediates and may or may not involve TE amplification [10, 11]. Class I TEs can be further subdivided into several orders; long terminal repeat (LTR) retrotransposons, non-LTR retrotransposons (sometimes also referred to as retroposons or long interspersed repetitive/nuclear elements (LINEs)), and Penelope-like elements (PLEs) [9, 10]. LTR retrotransposons share similarities with retroviruses, encoding a structural group-associated antigen (gag)-like protein, polymerase (pol)-like protein required for reverse transcription and genomic insertion, and sometimes a transmembrane receptor-binding envelope (env)-like protein, flanked by 200–500 bp regulatory non-translated LTRs [9–11]. LTR retrotransposons can be classified into four major clades, Ty1/copia, Ty3/gypsy, BEL and DIRS, based on their pol-encoded reverse transcriptase domain [10]. Non-LTR retrotransposons also encode a pol-like (ORF2) and sometimes a gag-like (ORF1) protein, and can be classified into 17 clades based on the pol-encoded reverse transcriptase domain [10]. Class II (DNA-mediated) TEs include ‘cut and paste’ DNA transposons, typified by 10–200 bp terminal inverted repeats (TIRs) that flank one or more ORFs encoding a transposase [9–11]. These elements transpose via a non-amplifying ‘cut and paste’ mechanism, with copy number increasing through cellular DNA repair mechanisms, and are classified into several families or superfamilies according to their transposase sequence [10]. The recently-discovered helitrons are thought to replicate via a rolling-circle mechanisms and encode proteins similar to helicases and replicases [9, 10]. Short interspersed elements (SINEs, class I) and miniature inverted repeat TEs (MITEs, class II) rely on proteins expressed from other TEs for their replication [9, 10] and would not be expected to be identifiable by PIT.

Characterising the proteomically active mobilome is of interest because TEs are implicated in processes as diverse as gene regulation [13, 14], mammalian pregnancy [15] and carcinogenesis [16]. TEs also contribute to genome plasticity and evolution [16, 17], and have been ‘domesticated’ for certain host functions [17]. Mobile genetic elements are of particular interest in relation to *Ae. aegypti*, because approximately 47% of the genome consists of TEs, contributing to a five-fold larger genome size compared to the distantly related mosquito *Anopheles gambiae* [8]. Furthermore, *Ae. aegypti* is a vector for several (re-)emerging arthropod-borne viruses (arboviruses), including dengue virus (DENV), the most significant arbovirus infecting humans [18], the re-emerging yellow fever virus (YFV), which continues to cause death and disease despite an effective vaccine [19], chikungunya virus (CHIKV), which has spread rapidly across the globe to become a major public health concern [20], and Zika virus (ZIKV), which recently emerged in the Americas and has been associated with a rise in microcephaly and neurological complications in Brazil [21]. Of relevance to arbovirus transmission, TEs have been shown to help establish persistent viral infections in mosquitoes and the model organism *Drosophila melanogaster* by reverse transcribing incoming viral RNA into extrachromosomal and/or genomically integrated DNA forms [22, 23]. Transcripts from these virus-derived genomic sequences feed in to the antiviral RNA interference (RNAi) pathway to suppress viral replication and allow viral persistence [23].

To our knowledge the mobilome-derived proteome has not been characterised for any organism. Previous studies have focussed on genes encoded by just a subset of transposons (such as transposase), and usually analysed a subset of spots on a 2D SDS-PAGE gel rather than performing a systematic proteomic analysis [24–31]. In mosquitoes, TE activity has additionally been inferred indirectly through comparative genomic and transcriptomic analyses [8, 32–34]. However, genomic data only provide a long-term evolutionary view of transposition events whilst transcriptomic data do not distinguish between *bona fide* TE activity and host responses mounted against TEs.

Here, we report for the first time a PIT analysis of a mosquito species, using the *Ae. aegypti* cell line Aag2. The proteins encoded by approximately 6,500 transcripts were identified, vastly increasing the number of experimentally confirmed *Ae. aegypti* proteins. The analysis provided 145 new genome annotations, the majority of which did not lie in either regions of poor sequence quality or mapping data, suggesting that the completeness of a genome’s assembly may not be a major driver behind gaps in annotation. The utility of PIT analysis for guiding annotation efforts was demonstrated by the identification of chromosome 1 and chromosomal loci 1p3, 1q4 and 2p4 as hotspots of poor annotation. We also detected 137 proteins expressed by TEs, representing the first proteomic characterisation of an organism’s mobilome. Interestingly, the relative abundance of specific mobile elements in the *Ae. aegypti* genome is not reflected at the protein level. While different types of genomic element are known to be more or less active in terms of transposition events in different cellular contexts [10, 16, 35–39], our proteomic analysis is the first to make this observation at the protein level, with clear implications for the co-opted functions of TE-derived proteins in cellular processes. The overrepresentation of long terminal repeat (LTR) retrotransposon proteins is of particular interest due to the potential role these TEs play in antiviral defence. Our data may inform future studies into virus persistence and arbovirus control methods, and demonstrate PIT’s utility for interrogating a genome’s annotation and proteomically active mobilome.

## Results

### Side-by-side comparison of PIT and ‘Conventional’ proteomic analysis of Aag2 cells

We performed PIT on Aag2 cells, an immortalised *Ae. aegypti* cell line commonly used for arbovirus research [40, 41]. Total RNA and protein was isolated from the same population of exponentially growing Aag2 cells. RNA-seq analysis of poly(A)^+^ enriched RNA resulted in ~73 million paired-end reads, 91 nt in length that were assembled into a *de novo* transcriptome using Trinity [42]. To maximise the protein search database for proteomic analysis, a combined database was prepared by translating each of the 73,881 Trinity transcripts and the official *Ae. aegypti* transcript list (Aedes-aegypti-Liverpool_TRANSCRIPTS_AaegL3.3.fa; 27,799 entries) in all 6 reading-frames (retaining ORFs > 200 nt) and combining the resulting protein sequences (62,675 and 53,824 sequences respectively) with the official *Ae. aegypti* predicted peptides list (Aedes-aegypti-Liverpool_PEPTIDES_AaegL3.3.fa; 17,703 entries) into a FASTA file. This file was then used as a search database for the MS/MS data acquired from analysis of the Aag2 proteome using MaxQuant/Andromeda [43, 44]. The analysis resulted in the identification of 6,124 unique protein groups (Fig. 1Bi), of which 5,215 were identified by two or more peptides (Fig. 1Bii). Although routinely only proteins identified by at least two peptides are reported in many studies, proteins identified using only one peptide using a stringent peptide-spectrum match (PSM) false discovery rate (FDR) can be informative [45, 46], especially when combined with specific transcript data [2, 3]. There was a good correlation between the protein groups identified using the *Ae. aegypti* translated transcripts search list and the predicted proteins list. The differences observed could reflect differences in the way the two databases are curated, i.e. protein predictions based on direct transcript analyses rather than from the reference genome sequence-derived transcript list, and/or the existence of alternative ORFs not present in the predicted peptides list. By contrast, the use of the translated Trinity transcriptome as a search database (‘PIT’ in Fig. 1B) resulted in 16% (Fig. 1Bi, all hits) and 14% (Fig. 1Bii, 2+ peptides) uniquely-identified protein groups (as a percentage of all identified protein groups) corresponding to all or 2 or more peptide hits respectively. The results confirm that PIT provides additional information compared to ‘conventional’ proteomics. A subset of protein groups were identified using the official *Ae. aegypti* transcript and predicted peptides lists but not by PIT, which has been reported before and may be due to insufficient RNA-seq coverage and/or incomplete *de novo* transcript assembly in this particular experiment [7].

### Characterisation of the Aag2 PIT proteome

To provide further information on the identity of protein groups uniquely identified by the PIT analysis, we performed a second PIT analysis of the *de novo* assembled transcriptome using a bespoke PIT workflow implemented in the Galaxy Integrated Omics (GIO) platform [7]. In this workflow, proteins identified by PIT were BLAST searched against the *Ae. aegypti*, *Culex quinquefasciatus*, *Drosophila melanogaster* and NCBI non-redundant protein RefSeq protein databases. *Ae. aegypti* and *Cx. quinquefasciatus* are distantly related mosquitoes classified in the order Diptera (flies) with the even more distantly related *D. melanogaster*. We extended our analysis to these non-*Aedes* insects to identify proteins that might be missing from the current *Ae. aegypti* genome assembly (AaegL3.3).

A total of 5,457 proteins (83% of the PIT proteome) matched known *Ae. aegypti* genes (Fig. 1Ci). Most of these (4,663) shared homology with *Cx. quinquefasciatus* proteins, and 2,231 matched proteins from all three dipteran insects, although we did not check whether these represent 1:1:1 orthologues. A further 215 hits (3% of the total) matched proteins from *Cx. quinquefasciatus* and *D. melanogaster*, but not *Ae. aegypti*. Their homology to non-*Aedes* insect proteins suggests these proteins represent *Ae. aegypti* genes not annotated in the current genome assembly (as described below). Finally, 14% of the PIT proteome (899 hits) shared no similarity with known *Ae. aegypti, Cx. quinquefasciatus* or *D. melanogaster* proteins. For simplicity, we will refer to these proteins as ‘non-insect hits’, although we acknowledge that the three dipteran species we used in our analysis do not encompass the full diversity of proteins expressed by all members of the class Insecta, and therefore a proportion of these hits might display homology to proteins from other insect species. These non-insect hits may represent agents such as viruses and TEs (see below). Overall, similar results were obtained when we included only proteins identified through two or more peptides in our analysis (Fig. 1Cii), although a larger proportion of the non-insect hits were identified with just one peptide. For the remainder of this report we used the complete list of identified proteins for our analyses unless otherwise indicated. As explained above, we believe that hits associated with one peptide still provide valuable biologically relevant information in the context of PIT, due to the additional transcriptomic evidence for their expression. The PIT-identified proteins are listed in Additional file 1.

### New annotation for the *Ae. Aegypti* reference genome

**Fig. 2:**
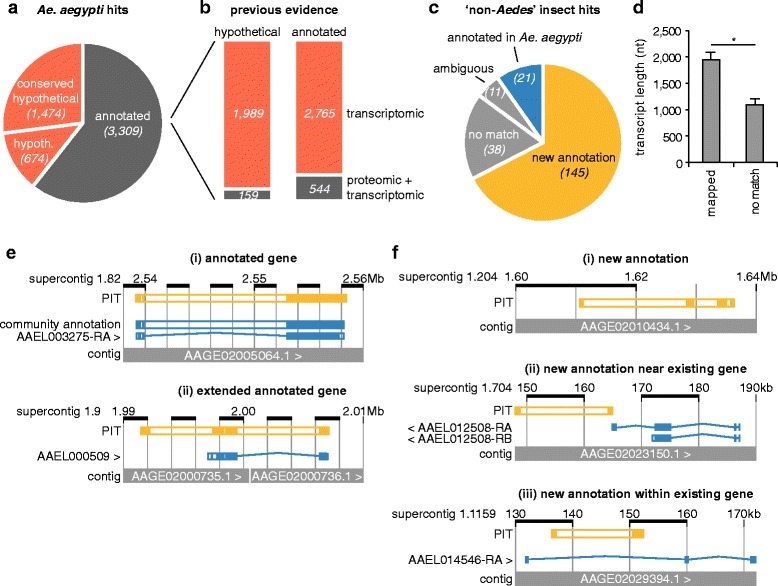
New annotation for the *Ae. aegypti* genome. **a** Vectorbase annotation of the PIT proteome that maps to *Ae. aegypti* (hypoth. denotes ‘hypothetical’ protein). **b** Previously published transcriptomic and proteomic evidence for the expression of our PIT-identified proteins that are either annotated or listed as ‘hypothetical’ or ‘conserved hypothetical’ (hypothetical) in Vectorbase (source data specified in Additional file 6). **c** Result of Vectorbase BLAST alignment against the *Ae. aegypti* genome of all 215 PIT transcripts initially marked as having homology to proteins from *Cx. quinquefasciatus* or *D. melanogaster*, but not *Ae. aegypti* (‘non-*Aedes*’ insect hits). **d** Mean transcript length of non-*Aedes* insect PIT hits that do (mapped) or do not (no match) map to the *Ae. aegypti* genome. Error bars represent standard error of the mean; * *P* < 0.001. **e** Examples of non-*Aedes* insect PIT transcripts mapping precisely (i), or as extensions or transcript variants (ii) of *Ae. aegypti* genes already annotated in Vectorbase (Trinity IDs 4627 and 1476 respectively). **f** Examples of non-*Aedes* insect PIT transcripts providing new genomic annotation as potentially novel ORFs (i to iii), or extensions or transcript variants of known ORFs (ii and iii) (Trinity IDs 3521, 1935 and 1124 respectively). (**d** and **e**) Contigs not shown in entirety; illustrations are modified Vectorbase BLAST alignments of representative PIT transcripts (images stylistically edited for clarity); <> indicates annotated transcript orientation; *filled boxes* represent regions of alignment to genome (exons)

Of the known *Ae. aegypti* proteins identified (Fig. 1Ci), 3,309 (61%) are annotated with names and/or functions (Fig. 2A). The rest are listed as ‘hypothetical’ (674 proteins, 12%) or ‘conserved hypothetical’ (1,474 proteins, 27%) (Fig. 2A). The proportion of annotated and ‘(conserved) hypothetical’ proteins in our data is comparable to their relative proportion in the reference genome [8]. All of these proteins had previously been verified at the transcript level, yet only a minority (703) have been detected at the protein level (Fig. 2B). The overrepresentation of annotated versus hypothetical proteins previously verified proteomically is possibly due to a bias towards proteins with better annotation in functional studies.

We next focussed on the proteins identified through homology to *Cx. quinquefasciatus* and *D. melanogaster*, but not *Ae. aegypti*, proteins (‘non-*Aedes* insect’ proteins) (Fig. 1Ci). We aligned their PIT transcripts to the *Ae. aegypti* reference genome (AaegL3.3) using the BLAST function at vectorbase.org [47] (Fig. 2C). Of the 215 non-*Aedes* insect proteins, 38 hits did not map to the *Ae. aegypti* reference sequence (Fig. 2C, no match). The short transcript length of these hits (Fig. 2D, no match) might reduce the ability of algorithms to detect homology across several shorter exons. A further 21 hits were already annotated, likely because of new annotation added since our initial PIT analysis (Fig. 2Ei). Eleven hits mapped to multiple locations in the genome (Fig. 2C, ambiguous) and were excluded from further analyses. These may represent duplicated genes due to genome assembly errors or paralogues arising through real gene duplication events. The remaining 145 hits (Fig. 2C) represent new annotation provided by PIT. This annotation takes several forms, such as extensions to predicted ORFs (Fig. 2Eii) that might be due to the current annotation being incorrect or because we identified an unknown transcript variant. We also identified new ORFs (Fig. 2Fi), and new exons close to or within existing ORFs (Fig. 2Fii and iii) that may represent new genes, or new transcript variants. Additional file 2 summarises our new annotation data.

### Evaluation of the *Ae. Aegypti* reference genome annotation using PIT

**Fig. 3:**
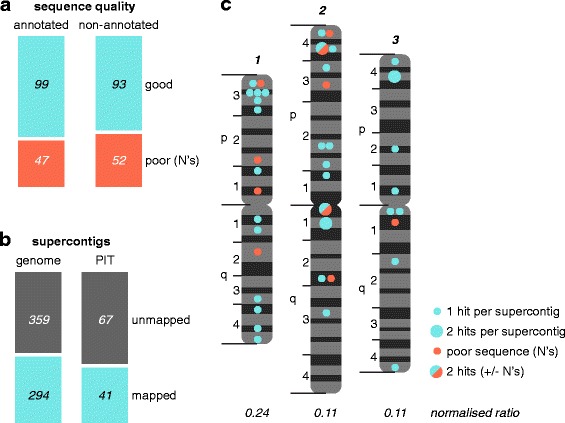
Interrogation of the *Ae. aegypti* genome annotation using PIT. **a** Sequencing gaps surrounding 145 previously non-annotated proteins identified by PIT (‘new annotation’ in Fig. 2C) compared to a matched sample of annotated *Ae. aegypti* genes (Additional file 7). **b** Number of supercontigs within the *Ae. aegypti* genome assembly, or the subset containing new annotation from PIT, that have been mapped to chromosomal locations [48]. **c** Supercontigs to which our PIT hits align mapped to the three *Ae. aegypti* chromosomes (map modelled on [48]). The normalised ratio of PIT-containing supercontigs to total mapped supercontigs per chromosome is also specified. Full mapping data given in Additional file 3

We next investigated why our new annotations were not already present in the *Ae. aegypti* reference genome. As a measure of sequence quality, we used vectorbase.org to analyse the genome sequences aligning to our PIT transcripts and the 5,000 up- and down-stream bases for sequencing gaps (N’s). Poor sequence quality was associated with 36% of our annotations (Fig. 3A, non-annotated). This was slightly higher than, but comparable to, a set of randomised PIT transcripts mapping to annotated genes (Fig. 3A, annotated).

When the *Ae. aegypti* genome was sequenced, sequencing reads were assembled into contigs and then supercontigs and scaffolds [8]. Some of these supercontigs have been assigned physical chromosomal locations [8, 48–50]. We used the most recent mapping data [48] as a proxy for the quality of the genome assembly surrounding our PIT annotation. Chromosomal locations have been assigned to 45% of all supercontigs, compared to 38% of supercontigs containing new PIT annotation (Fig. 3B). Note that some supercontigs contain more than one PIT hit. Although there is a trend for our PIT annotation to map to regions of poor sequence (Fig. 3A) and assembly (Fig. 3B) quality, this trend is not pronounced, suggesting the completeness of a genome’s assembly may not be a major driver behind gaps in annotation.

The 42 supercontigs containing new annotation were spread across all three chromosomes (Fig. 3C). Normalised to the number of supercontigs mapped to each chromosome, new PIT annotation exhibited a two-fold enrichment on chromosome 1. Normalised on a finer scale, chromosomal regions 1p3, 1q4 and 2p4 were enriched in new annotation. While annotation on 1p3 and 2p4 was associated with poor sequence quality, annotation on 1q4 was not. Full mapping data is given in Additional file 3. Our data demonstrate that PIT can evaluate the state of a genome’s annotation by identifying hotspots of incomplete annotation.

### Identification of proteins derived from mobile genetic elements

We next turned to the PIT hits lacking homology to *Ae. aegypti, Cx. quinquefasciatus* or *D. melanogaster* proteins (Fig. 1Ci, “non-insect”). We reasoned that some of these hits may stem from transposons or viruses. Studying the proteome derived from highly repetitive mobile genetic elements is intrinsically difficult for several reasons. Individual elements may be present in multiple copies within the genome, and each copy may differ markedly from the consensus sequence [51]. Elements that invaded the genome more recently are less divergent from their consensus sequence [9, 51]. However, the repertoire of recently acquired elements differs between individuals in both the identity and genomic location of TEs [51, 52]. Therefore, a given experimental sample may match poorly to the organism’s reference genome. The sequence of evolutionarily ancient invaders, on the other hand, may degenerate until these TEs are difficult to identify [9, 51, 53]. While ancient TEs are less likely to be actively transposing [51], they may still express protein, especially where a TE protein is co-opted for host cell functions [17]. Finally, repetitive genetic elements are masked in genome reference sequences and therefore do not show up in classical transcript and protein databases [53].

For these reasons, proteomic studies of the mobilome have so far focussed on a subset of TE proteins, such as transposase [24, 25], and are therefore inherently biased and non-systematic in their approach. We hypothesised that PIT might overcome some of the intrinsic difficulties in studying the mobilome, because PIT allows potentially divergent TE proteins to be matched to their corresponding transcripts irrespective of differences from the reference genome. *Ae. aegypti* provides a tractable platform for a proof-of-principle study because a large number of mobile genetic elements previously identified in evolutionarily divergent mosquito species have been curated in the TEfam database (tefam.biochem.vt.edu). While it is likely that many more TEs remain to be identified and characterised in each of these mosquito species, the existing data allowed us to validate our approach.

We began by BLASTing the translated ORFs from the Trinity transcript list that had been experimentally confirmed by PIT, and had been classified as ‘non-insect’ hits, against all mosquito TE proteins in the TEfam database. In principle, any (non-PIT) proteomic dataset can be searched by BLAST against the TEfam database. However, given the highly divergent nature of mobile genetic elements, the *in silico* translated transcripts (which were experimentally matched with peptides) should identify more low homology hits than the much shorter peptides identified by LC-MS/MS. Identifying these lower homology matches is essential for characterising proteomically active TEs that diverge markedly from the limited list of known mobile genetic elements.

**Fig. 4:**
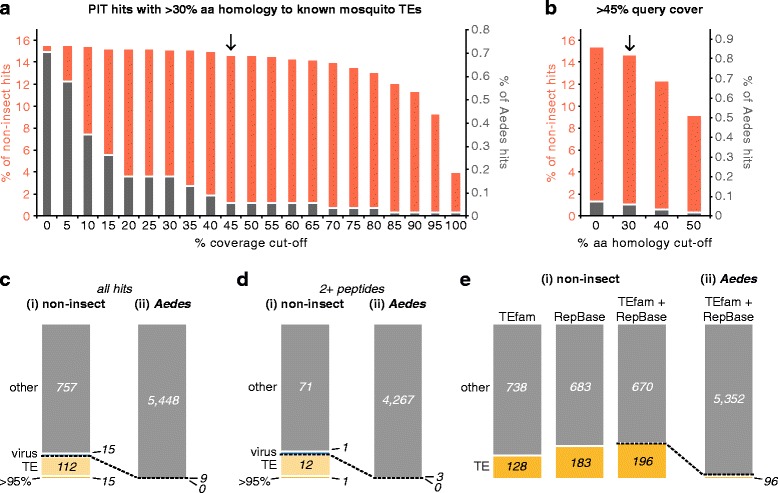
Identification of proteins derived from mobile genetic elements using PIT. **a** Proportion of non-insect PIT hits (*red*) and PIT hits matching known *Ae. aegypti* genes (*grey*) that display >30% amino acid sequence similarity to known mosquito TEs (E-value <10^5^) at increasing thresholds for % sequence coverage. *Arrow* indicates optimal threshold for TE identification, used for all remaining analyses unless otherwise specified. **b** As for (**a**), except that the proportion of PIT hits with >45% sequence coverage is plotted at different sequence similarity thresholds. **c** (i) Breakdown of the non-insect PIT proteome into virus-derived proteins, proteins with homology to known mosquito TEs (Additional file 4), and other non-classified proteins (other). Proteins with >95% amino acid homology and >95% sequence coverage were considered ‘exact matches’ to known TEs (Table 1). (ii) Identification of TEs in the *Aedes* PIT proteome. **d** as for (**c**), except only hits associated with two or more peptides were included in the analysis. **e** (i) Identification of TEs in the non-insect PIT hits using either TEfam, RepBase, or both databases as a reference. (ii) Identification of TEs in the *Aedes* PIT proteome using a combined TEfam and RepBase reference database

Given the lack of high-throughput proteomic studies on TEs, we then sought to empirically define criteria for identifying TE proteins with high confidence. Using an E-value threshold of 10^−5^, considered a significant match at the amino acid level, we identified 149 proteins matching TEs from *Ae. aegypti*, *Cx. quinquefasciatus* and *Anopheles spp.* (17% of all non-insect hits). We next used the PIT dataset corresponding to known *Ae. aegypti* genes as a calibrator for determining a stringent, yet inclusive, threshold for TE protein identification. Since repetitive mobile genetic elements are masked in the reference genome [8], we would expect our *Ae. aegypti* PIT hits to contain no TE proteins. However, in practice a small number of TE-derived proteins might be included in the reference genome if they are misannotated or have been co-opted for known cellular functions. At the level of amino acid homology, 90% of protein pairs with 30% identity or greater represent *bona fide* homologous proteins [54]. While TE proteins might be expected to be more divergent, we used this threshold to plot PIT hits matching known TE proteins in the non-insect and *Ae. aegypti* datasets at different % amino acid sequence coverage cut-offs (Fig. 4A). As expected, the *Ae. aegypti* dataset contained a lower proportion of hits with homology to TE proteins (Fig. 4A). At a cut-off of 0% coverage (i.e. all identified hits) 15.5% of the non-insect hits matched TE proteins, while only 0.7% of *Ae. aegypti* hits did (Fig. 4A). As the % coverage cut-off is increased, the number of TE matches drops rapidly in the *Ae. aegypti* dataset (Fig. 4A), suggesting that a large proportion of the TE hits mapping to *Ae. aegypti* genes are false positives. In contrast, the TE hits in the non-insect dataset are predominantly resistant to an increase in the % coverage cut-off (up to about 75% sequence coverage), suggesting that the majority of the TE proteins identified in this dataset represent real TE proteins. Therefore, our criteria for the identification of TE proteins was >30% amino acid identity across >45% sequence coverage, which maximises the number of *bona fide* TE proteins identified in the non-insect database, while minimising the number of potential false positives in the *Ae. aegypti* PIT dataset (Fig. 4A). At >45% sequence coverage, increasing the % identity threshold has a marginal impact on ‘background’ TE detection in the *Ae. aegypti* PIT list, while the number of real TE proteins identified in our non-insect dataset is reduced (Fig. 4B). Therefore, the lower 30% identity threshold is preferable for maximising the detection of *bona fide* TE proteins.

Using these criteria, we identified 127 TE proteins in our non-insect PIT hits (14.1% of the total) (Fig. 4Ci). On the other hand, just nine (0.2%) of *Ae. aegypti* PIT hits matched TE proteins (Fig. 4Cii). Additional file 4 lists the complete set of TE proteins identified by PIT. Fifteen non-insect proteins exhibited >95% amino acid homology to known *Ae. aegypti* TEs across >95% sequence coverage (Fig. 4Ci, Table 1), which, given the highly divergent nature of mobile genetic elements, might be considered the closest thing to an ‘exact match’ [12]. The remaining TE proteins shared lower homology with known mobile elements from *Ae. aegypti* and other mosquito species. Importantly, the overall proportion of TE proteins identified in the non-insect and *Ae. aegypti* PIT datasets did not change when we analysed only PIT-identified proteins associated with two or more peptide hits (Fig. 4D). In addition to using TEfam as a source database for identifying TE proteins, we also used RepBase (girinst.org), a larger database of TEs from eukaryotic genomes [12]. Using RepBase alone, or in combination with TEfam, increased the number of TE proteins identified in our non-insect dataset by up to 53% (Fig. 4Ei). Importantly, almost all of the TE proteins identified by TEfam alone (Fig. 4Ci, Additional file 4) were also identified using RepBase, with close correlation in terms of the type of elements identified, indicating that these TE proteins are high-confidence hits. Of the new TE proteins identified through the RepBase database (which also matched TEfam proteins below our thresholds for inclusion) 41% disagreed with TEfam in terms of the TE identity (LTR retrotransposon versus cut and paste DNA transposon, etc.), with only 25% agreeing at the clade/superfamily level. Since these hits may therefore be of lower quality, we decided to focus on our more robust hits identified in the initial TEfam analysis (Fig. 4Ci, Additional file 4) for the remainder of this paper. In fact, we found that combining TEfam and RepBase as source databases for the identification of TE proteins increased the ‘background’ identification of TE proteins in our *Aedes* protein dataset (Fig. 4Eii). Interestingly, 96% of TE proteins identified through RepBase alone matched mosquito TEs, suggesting that TEfam, which is more tailored to mosquito TEs and identified highly confident hits in our analysis, may be sufficient and favourable for the identification of mosquito TEs over RepBase.

**Table 1:** Known *Ae. aegypti* TEs Active at the Protein Level in Aag2 Cells

TE Order	TE Clade	Element	ORF Detected	TEfam ID
LTR	Ty3/gypsy	Ele7	pol	TF000099
		Ele19	gag	TF000110
		**Ele40**	**gag**	**TF000135**
		Ele50	env-like	TF000145
		Ele54	env-like	TF000317
		Ele55	env-like	TF000318
		Ele69	gag	TF000341
		Ele101	gag	TF000382
		Ele104	pol	TF000385
		Ele122	env-like	TF000419
		Ele154	gag	TF000540
		Ele227	gag	TF000507
	BEL	Ele153	gag	TF000298
Non-LTR	R4	Ele1	ORF1	TF000040
	Jockey	Ele1	ORF2	TF000019

All elements are class I TEs. Detected proteins exhibit >95% amino acid homology and >95% sequence coverage with specified elements

Bold typeface indicates Trinity transcripts associated with two or more peptides

In summary, our analysis demonstrates that the use of PIT with the criteria we have described can identify proteins expressed from mobile genetic elements in an organism’s genome. Furthermore, PIT is superior to ‘conventional’ proteomic approaches in this regard, as the vast majority of TEs were identified in the ‘non-insect’ list of hits that were only identified by PIT.

Using our thresholds for TE identification, a number of non-insect hits (772) remained unidentified (Fig. 4Ci, other). Whilst many of these hits might represent TEs that fall below our threshold of detection, we also identified proteins derived from the insect-specific flavivirus cell fusing agent virus (CFAV), a known contaminant of Aag2 cells, and the recently discovered insect-specific bunyavirus Phasi Charoen-like virus (PCLV), which we identify here as a previously unknown contaminant of Aag2 cells (Fig. 4Ci and Di, virus, and Additional file 5). We confirmed that PCLV is an actively replicating virus in Aag2 cells (Additional file 5), and deposited all viral genome sequences in Genbank (accession numbers KU936054, KU936055, KU936056 and KU936057). For more information on these viruses, please refer to Additional file 5. The remaining 757 non-insect hits (Fig. 4Ci, other) showed sequence identity to TEs which was below our cut-off threshold value and may be explained by; erroneous assembly of transcripts by Trinity, false assignment of specific peptides to a protein predicted from the transcriptome, the presence of contaminants and/or poor annotation of specific TEs in the sequence databases.

### Types of mobile elements expressing protein in Aag2 cells

**Fig. 5:**
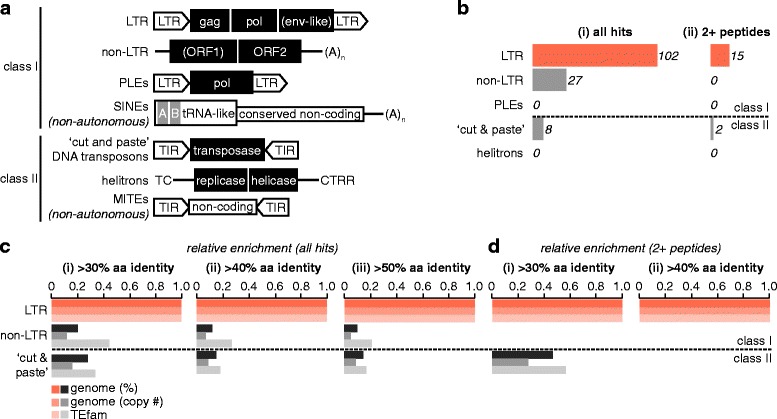
LTR retrotransposons are disproportionately active at the protein level compared to other TEs. **a** Schematic illustrating representative TEs previously identified in mosquitoes (not to scale). *Filled boxes* indicate protein-coding regions, *open boxes* and *grey shading* indicate non-translated regions and conserved non-coding domains (*grey*). Note that not all SINEs are tRNA-like. Bracketed ORFs are not always found in that TE subclass. Typical terminal amino acids are indicated for non-LTR retrotransposons, SINEs and helitrons. Env-like, envelope-like protein (incomplete); gag, group antigen; LTR, long-terminal repeat retrotransposons; MITE, miniature inverted repeat transposable element; non-LTR, non-LTR retrotransposon; PLE, Penelope-like element; pol, polymerase; SINE, short interspersed element; TIR, terminal inverted repeat. **b** Absolute abundance of proteins derived from known mosquito protein-coding TEs in the total PIT proteome (i) and for all PIT hits associated with two or more peptides (ii). **c** Relative enrichment of proteins from detected TEs using >30% (i), >40% (ii) and >50% (iii) amino acid identity as a threshold for TE discovery (>45% sequence coverage throughout). Enrichment is shown relative to the proportion of the *Ae. aegypti* reference genome that is comprised of sequences derived from each respective TE subclass (genome (%)) [8], relative to the total copy number of elements from each TE subclass within the *Ae. aegypti* reference genome (genome (copy #)) [8], and relative to the total number of entries for each TE subclass in the TEfam database used to identify TEs in our dataset. Enrichment is shown relative to LTR retrotransposons. **d** as for (**c**), except that only PIT hits identified through more than two peptides are shown

We next wanted to determine what sorts of TEs express protein in *Ae. aegypti* cells. Mobile genetic elements previously described in mosquitoes are summarised in Fig. 5A. We found proteins from LTR retrotransposons, non-LTR retrotransposons and ‘cut and paste’ DNA transposons in our complete PIT dataset, with a larger number of LTR retrotransposon proteins detected compared to other TEs (Fig. 5Bi, Additional file 4). A larger number of LTR retrotransposon proteins was also detected when only TE proteins identified through two or more peptides were analysed, although in this case we were no longer able to detect proteins derived from non-LTR retrotransposons (Fig. 5Bii). This overrepresentation of LTR retrotransposons could in principle result from a greater number of LTR retrotransposons in the TEfam database, which includes TEs from non-*Aedes* mosquitoes, or in the *Ae. aegypti* genome (both in terms of absolute sequence coverage and the number of elements present). We therefore normalised the absolute number of proteins detected in our PIT dataset for each type of TE to the respective known representation of each type of element in the TEfam database or *Ae. aegypti* reference genome, and plotted the resulting ‘relative enrichment’ proportional to the relative abundance of LTR retrotransposons, which was set to 1 (Fig. 5Cand D). Interestingly, LTR retrotransposon proteins remained enriched two- to nine-fold (Fig. 5Ci), while proteins from non-LTR retrotransposons and ‘cut and paste’ DNA transposons were detected at similar relative frequencies to each other despite differences in their absolute abundance (Fig. 5Bi). To exclude the possibility that this observation was an artefact of our thresholds for TE identification, we repeated our analysis using only TE proteins exhibiting >40% or >50% amino acid identity to known mosquito TE proteins (at >45% sequence coverage throughout) (Fig. 5Cii and iii). At these higher thresholds, the ‘background’ identification of proteins derived from mobile genetic elements in our *Ae. aegypti* PIT dataset is reduced, although many *bona fide* TE proteins are likely also excluded (Fig. 4B). We observed an even greater four- to 19-fold enrichment of LTR retrotransposon proteins in this more stringent analysis (Fig. 5Cii and iii). Finally, we repeated our analysis using only hits associated with more than two peptides (Fig. 5D). Again, LTR retrotransposon proteins remained overrepresented in our PIT data (Fig. 5D), and were the only elements identified with greater than 40% amino acid identity to known TE proteins (Fig. 5Dii). We conclude that, under our experimental conditions, LTR retrotransposons are more likely to express protein at levels detectable by PIT than other *Ae. aegypti* mobile genetic elements.

**Fig. 6:**
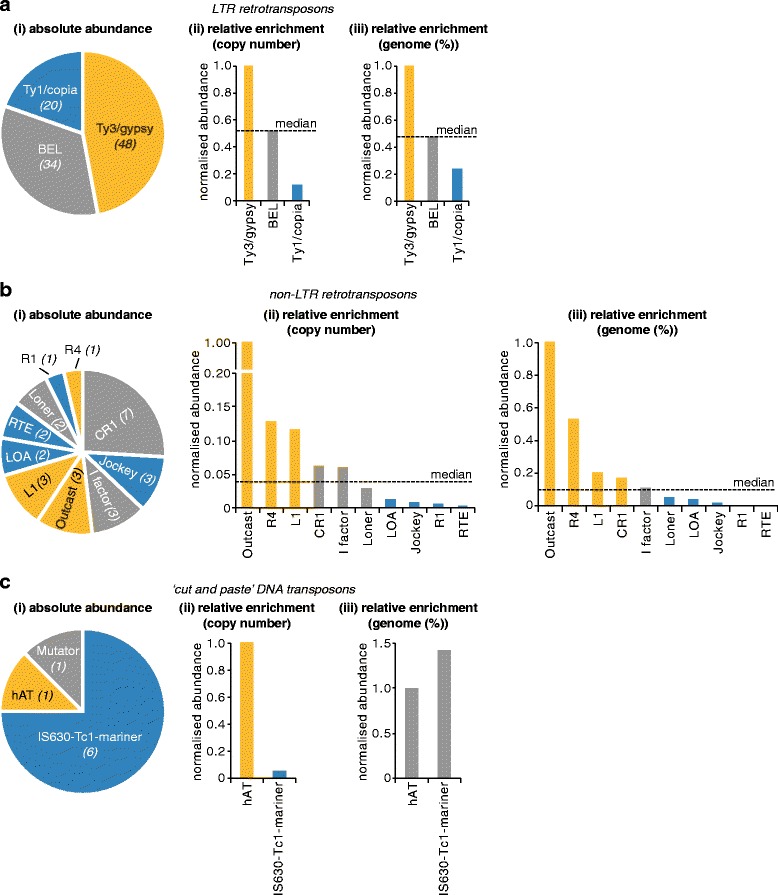
Protein expression from mobile genetic elements does not correlate with their genomic abundance. **a-c** Breakdown of elements from different TE clades/superfamilies detected in the total PIT proteome. (i) absolute number (clockwise in decreasing order of abundance), (ii) relative enrichment compared to copy number in the *Ae. aegypti* genome [8], (iii) relative enrichment compared to *Ae. aegypti* genome coverage (%) [8]. *Yellow*, overrepresented TEs; *blue*, underrepresented TEs. Genome copy number was not known for Mutator (**c**)

The data were then analysed at the level of TE clades/superfamilies. For LTR retrotransposons, we detected more proteins from Ty3/gypsy and BEL elements than Ty1/copia elements (Fig. 6Ai). The relative enrichment of Ty3/gypsy proteins and underrepresentation of Ty1/copia proteins was still observed when normalised to their respective copy number (Fig. 6Aii) or % coverage (Fig. 6Aiii) in the *Ae. aegypti* genome. This discrepancy between an element’s genomic abundance and the detection of its proteins is more clearly illustrated for non-LTR retrotransposons (Fig. 6B), which are classified into more clades than LTR retrotransposons. For example, the second highest number of detected proteins come from Jockey elements, which are nevertheless underrepresented relative to their genomic abundance, while the elements L1 and Outcast are detected in lower absolute numbers, but are overrepresented relative to their genomic abundance (Fig. 6Bi, ii and iii). The data for ‘cut and paste’ DNA transposons are unclear, since IS630-Tc1-mariner elements were underrepresented relative to genome copy number but not % genome coverage (Fig. 6C). We did not detect protein from L2 (non-LTR retrotransposon) or P, piggyBac, PIF-harbinger or Transib (‘cut and paste’ DNA transposons) elements, even though some of these TEs are present in higher numbers in the TEfam database and *Ae. aegypti* genome than other detectable elements. Therefore, at different classification levels, the abundance of mobile elements in a host’s genome does not correlate with their activity at the protein level.

### TE ORFs expressed in Aag2 cells

**Fig. 7:**
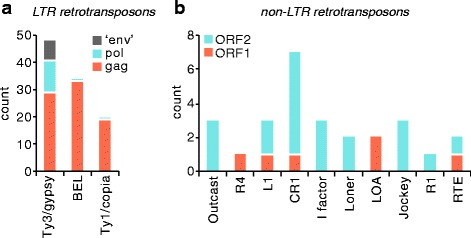
Protein expression across ORFs for TEs that encode multiple ORFs. **a** LTR retrotransposons and **b** non-LTR retrotransposons

Of the TEs for which we detected protein, LTR and non-LTR retrotransposons encode more than one ORF (Fig. 5A). For all LTR retrotransposon clades, we detected a greater number of gag proteins than pol proteins (Fig. 7a). Protein from env-like genes was only detected for a few Ty3/gypsy elements. Note that this analysis quantifies the total number of different elements for which each ORF was detected, not absolute TE protein levels. For non-LTR retrotransposons, the reverse trend was observed, with more proteins detected for ORF2 than ORF1 (Fig. 7b). Of the 15 ‘perfect match’ TEs (Fig. 4Ci, Table 1), there were none for which more than one ORF was detected. We therefore conclude that TE ORFs are not expressed equally at the protein level in Aag2 cells.

## Discussion

We report, for the first time, a PIT analysis of a mosquito species, and provide the first protein expression data for almost a third of *Ae. aegypti* genes. This includes 1,989 (27% of) genes listed as ‘(conserved) hypothetical’ and lacking functional annotation. We provide 145 new annotations, representing novel genes and new exons for known transcripts. Our data will benefit molecular studies, where misannotations waste time and resources. Intron-exon junctions in particular are incorrectly annotated in many genome assemblies [53], and we confirm that PIT can help resolve these (Fig. 2E) [55–57].

While PIT has been used to provide annotation in other organisms [56–58], this study is the first to demonstrate PIT’s utility in interrogating the state of a genome’s annotation. By its very nature, this sort of proteomic analysis cannot be performed when the reference genome is used to identify peptides. We also establish proof-of-principle that PIT can characterise the proteome derived from an organism’s mobile genetic elements, and show that PIT identifies vastly more elements compared to ‘conventional’ proteomics analyses that rely on existing genome annotation.

### PIT as a tool for evaluating a Genome’s annotation

The reference genomes of non-model organisms often lack comprehensive annotation, with missing, out-of-frame and duplicated ORFs, incorrect splicing predictions and poor functional annotation, posing problems for molecular studies [1, 53, 56, 57]. Given limited resources, identifying hotspots of incomplete annotation would focus annotation efforts and maximise their impact. By identifying *Ae. aegypti* chromosome 1, and chromosomal regions 1p3, 1q4 and 2p4, as enriched in new PIT annotation, we demonstrate PIT’s utility in characterising such hotspots. PIT is superior to proteomic or transcriptomic analyses alone, as PIT simultaneously verifies protein expression and an ORF’s transcript structure. Although chromosomal regions 1p3 and 2p4 were associated with poor sequence quality, region 1q4 was not, and more generally our new annotations were only marginally associated with poor sequence and assembly quality. This suggests that the completeness of a genome’s assembly may not be a major driver behind gaps in annotation. Instead, the large number of minisatellites and rapid evolution of chromosome 1 [48] may hinder gene prediction and annotation [53]. Furthermore, some PIT annotations were close to contig termini (data not shown), and genes spanning misassembled contigs often lack annotation [56]. Regions with low manual annotation are also enriched for new PIT annotation [56]. We mapped new annotation to chromosomal locations, but where this is not possible a list of incompletely annotated contigs, as we also provide (Additional file 3), is still of benefit.

### PIT for characterising the proteome expressed by an organism’s mobilome

We present the first characterisation of an organism’s proteomically active mobilome. Only 15 proteins exactly matched known *Ae. aegypti* TEs. This might reflect mobilome differences between mosquito populations [52] and Aag2 cells not captured in the single existing reference genome. TE sequences are also difficult to characterise [53], perhaps leading to incomplete TE annotation. In fact, at least 122 mobile genetic elements were identified that are not represented in the TEfam database, and the majority of these hits map with close to 100% homology to non-annotated regions of the *Ae. aegypti* reference genome (data not shown), suggesting that the current list of *Ae. aegypti* TEs is incomplete. Our data demonstrate the utility of PIT in the discovery and annotation of protein-expressing mobile elements. Of note, Mutator, a ‘cut and paste’ DNA transposon, was identified for the first time in *Ae. aegypti*.

For this initial proof-of-concept study, a threshold for TE protein identification was empirically determined that we believe to be highly stringent, yet inclusive. However, it was noted that almost all (non-viral) PIT hits in our non-insect dataset displayed varying levels of homology to known mosquito mobile genetic elements, which was not the case for the *Ae. aegypti* PIT hits (data not shown). Given the highly divergent nature of mobile genetic elements, and their likely incomplete representation in the TEfam database, many more of these hits may represent *bona fide* TE-derived proteins. By compiling a database of *Ae. aegypti* TE proteins identified by PIT in different cell lines and wild and laboratory mosquitoes under varying experimental conditions it should in the future be possible to more confidently characterise a larger array of mobile genetic elements in this mosquito species.

### Genomic abundance does not predict a TE’s protein expression

Interestingly, at different levels of classification, the genomic abundance of mobile genetic elements in Aag2 cells did not predict protein expression. Similar observations regarding the propensity for a transposon to transpose have been made previously [10, 16, 35–39]. However, at the level of expression of individual TE proteins, this observation could not have been predicted prior to the systematic analysis of the proteomically active mobilome reported here. This distinction is important because many non-transposing genomic elements might be contributing to cellular functions through the expression of their individual proteins, and we demonstrate that PIT is particularly well-suited to further elucidating such non-transposition-related functions of TEs. Interestingly, we found LTR retrotransposon proteins to be overrepresented compared to other types of mobile genetic elements (Fig. 5Cand D). At the clade/superfamily level, a similar discordance between the genomic abundance of elements and their protein expression was observed (Fig. 6). While half of the *Ae. aegypti* genome consists of TEs, only around 200 encode intact ORFs [8]. At the level of individual TEs, protein expression was only detected from 15 of these elements (Fig. 4Ci, Table 1). Despite the likelihood of incomplete proteomic coverage, it would therefore appear that not all elements encoding intact ORFs express protein. Finally, not all proteins are equally detectable proteomically for TEs encoding multiple ORFs (Fig. 7). For elements that exactly match known *Ae. aegypti* TEs, expression of more than one ORF per element could not be detected.

Although incomplete PIT coverage and the unknown abundance of elements in the Aag2 genome might influence our results, this was compensated for by also normalising to the TEfam database. Given the large number of data points at the class and clade/superfamily level, we believe our overarching conclusions hold true, even though trends might change for individual elements. Additionally, the enrichment of LTR retrotransposon proteins (Fig. 5Cand D) correlates with the overrepresentation of small RNAs derived from these elements, which predict TE activity [59], in *D. melanogaster* [60].

Several mechanisms might account for the lack of correlation between TE protein expression and the presence of an element/ORF in the genome. Firstly, genomic elements themselves regulate their own protein expression [14]. Secondly, host immune pathways suppress TE activity to protect against the deleterious genomic effects of transposition, and it is conceivable that certain elements and their ORFs are more effectively silenced by host immunity [17, 51]. Finally, a subset of TE proteins may be co-opted for host cell functions [17], while other ORFs within the same element, and other elements within the same clade/superfamily, may not be active in this way.

### Biological implications of TE protein expression detected by PIT

Our data cannot prove transposition-in-progress. Instead, the strengths of PIT lie in its ability to provide an unbiased global characterisation of protein expression from mobile genetic elements, whether or not these are in the process of actively transposing. PIT is therefore useful as a starting point for functional studies into transposon activity and TE proteins co-opted for cellular functions. PIT can also give insight into mobilome biology. Non-LTR retrotransposons for example are often 5’-truncated due to incomplete reverse transcription during transposition [61], which is reflected in the higher abundance of ORF2 versus ORF1 proteins in our dataset (Fig. 7b). The greater abundance of gag proteins than env-like proteins of LTR retrotransposons also makes sense, because, unlike endogenous retroviruses, not all LTR retrotransposons encode an env-like protein [9, 10]. In addition, gag is a structural component of the virus-like particles produced during the lifecycle of LTR retrotransposons, and therefore more gag is required (and produced) than the enzymatically active non-structural pol protein [62]. These observations highlight the ability of PIT to provide biologically relevant information about TEs.

For arbovirus transmission, the overrepresentation of LTR retrotransposon proteins is of particular interest. These TEs are enriched at genomic sites of viral sequence integration and play a critical role in arbovirus persistence in insects [23]. Cells may distinguish between such ‘useful’ elements and ‘hostile’ TEs [61]. It is unclear whether LTR retrotransposon protein expression is tolerated to facilitate mosquito immunity, or whether these elements are better at evading host defences against TEs. Our data provide a starting point for studies into how specific LTR retrotransposons contribute to arboviral persistence and transmission. A list of proteomically active TEs should also facilitate the identification of active elements for typing mosquito populations, and might improve transposon-mediated genome editing of mosquitoes [10]. Given that our current dataset is representative of just one experimental condition in one cell line, it will be important in the future to expand on these proof-of-principle studies by analysing different experimental conditions and by expanding our work into wild and laboratory mosquito populations.

## Conclusions

PIT represents a valuable new tool to investigate mobilome activity and protein expression, since genomic data cannot identify current mobilome activity and RNA-seq data do not distinguish between TE activity and cellular defences against TEs [32, 63, 64]. Furthermore, our data on TE protein expression, *Ae. aegypti* genome annotation and persistent Aag2 viruses are of value to arbovirus-vector interaction studies. Finally, we provide proof-of-principle for PIT’s usefulness in evaluating genome annotation, with clear utility in guiding annotation efforts in the increasing number of sequenced genomes of non-model organisms.

## Methods

### Cells

*Ae. aegypti* Aag2 cells [41, 65] were a kind gift from Alain Kohl (University of Glasgow, UK) and Raul Andino (University of California, San Francisco, CA USA), and were maintained in Leibovitz’s L-15 medium (ThermoFisher Scientific, Waltham, MA USA) supplemented with 2 mM glutamine, 0.1 mM non-essential amino acids, 8% (v/v) tryptose phosphate broth (Sigma-Aldrich, St. Louis, MO USA), 100 U/ml penicillin, 100 μg/ml streptomycin and 10% (v/v) foetal bovine serum (FBS) (ThermoFisher Scientific) at 28 °C in a humidified atmosphere without CO_2_.

### RNA and protein purification from Aag2 cells

Approximately 2 × 10^7^ Aag2 cells (grown to 90% confluency) were scraped into the culture medium, harvested by centrifugation, washed twice with ice cold PBS and split into two samples which were then used for either RNA or protein extraction. For RNA isolation, the cell pellet was resuspended in 1 ml of TRIzol® reagent (ThermoFisher Scientific) and purified as described by the manufacturer. The purified RNA then underwent a second round of purification using an RNeasy MinElute spin column (Qiagen, Venlo, Netherlands). For protein extraction, the cell pellet was suspended in 0.5 ml of 2X Laemmli sample buffer (without bromophenol blue) and heated to 95 °C for 5 min. The protein concentration in the cell lysate was determined using a BCA Protein Assay kit (ThermoFisher Scientific).

### RNA-seq analysis

A sample of the Aag2 total RNA was supplied to the Beijing Genomics Institute (Beijing, China) for Eukaryotic RNA-seq (Transcriptome) analysis which entailed; RNA integrity analysis, poly(A)^+^ enrichment and cDNA library production, followed by sequencing using an Illumina HiSeq2000. After filtering, the analysis resulted in a dataset containing ~73 million paired-end reads 91 bp in length. The sequencing data was then uploaded to the Galaxy suite of software, hosted locally on BlueCrystal, the University of Bristol High Performance Computer. The Trinity *de novo* assembly software [42], hosted locally on Galaxy, was used to produce a set of assembled transcripts from the RNA-seq data (73,881 entries) using the default parameters. The RNAseq data is available at the European Nucleotide Archive with the accession number PRJEB13078 (http://www.ebi.ac.uk/ena/data/view/PRJEB13078).

### LC-MS/MS analysis

The proteins in 50 μg of total protein extract were separated by 10% SDS-PAGE. The gel lane was cut into 20 slices and each slice subjected to in-gel tryptic digestion using a ProGest automated digestion unit (Digilab, Marlborough, MA USA). The resulting peptides were fractionated using a Dionex Ultimate 3000 nanoHPLC system in line with an LTQ-Orbitrap Velos mass spectrometer (ThermoFisher Scientific). In brief, peptides in 1% (v/v) formic acid were injected onto an Acclaim PepMap C18 nano-trap column (Dionex, Sunnyvale, CA USA). After washing with 0.5% (v/v) acetonitrile 0.1% (v/v) formic acid, peptides were resolved on a 250 mm × 75 μm Acclaim PepMap C18 reverse phase analytical column (Dionex) over a 150 min organic gradient, using 7 gradient segments (1–6% solvent B over 1 min, 6–15% B over 58 min, 15–32% B over 58 min, 32–40% B over 3 min, 40–90% B over 1 min, held at 90% B for 6 min and then reduced to 1% B over 1 min) with a flow rate of 300 nl min^−1^. Solvent A was 0.1% formic acid and Solvent B was aqueous 80% acetonitrile in 0.1% formic acid. Peptides were ionized by nano-electrospray ionization at 2.3 kV using a stainless steel emitter with an internal diameter of 30 μm (ThermoFisher Scientific) and a capillary temperature of 250 °C. Tandem mass spectra were acquired using an LTQ-Orbitrap Velos mass spectrometer controlled by Xcalibur 2.1 software (ThermoFisher Scientific) and operated in data-dependent acquisition mode. The Orbitrap was set to analyze the survey scans at 60,000 resolution (at m/z 400) in the mass range m/z 300 to 2000 and the top six multiply charged ions in each duty cycle selected for MS/MS in the LTQ linear ion trap. Charge state filtering, where unassigned precursor ions were not selected for fragmentation, and dynamic exclusion (repeat count, 1; repeat duration, 30 s; exclusion list size, 500) were used. Fragmentation conditions in the LTQ were as follows: normalized collision energy, 40%; activation q, 0.25; activation time 10 msec; and minimum ion selection intensity, 500 counts. Data are available via ProteomeXchange with identifier PXD003799.

### Proteomic analysis

PIT analysis was done using a bespoke bioinformatic pipeline (PIT: Genome annotation_from mgf; http://gio.sbcs.qmul.ac.uk/root?workflow_id=63cd3858d057a6d1) available on the publically available proteomics resource GIO [7]. The default settings on each tool contained in the pipeline were used unless otherwise stated, as follows. The 20 .RAW files from the MS/MS analysis were first converted to mzML files using MSConvert whilst the *de novo* transcriptome produced by Trinity (containing 73,881 sequences) was translated in all 6 frames (ORFs with a start codon > 200 nt) using PIT:ORFall to produce 62,675 ORFs. The resultant mzML and FASTA files were used for a database search and subsequent downstream processing using MSGF + MSMS Search and PIT:PSMProcessing. Cysteine carbamidomethylation was set as a fixed modification and methionine oxidation and N-terminal acetylation as variable modifications in the search. Searches were performed with full tryptic digestion, a MS tolerance of 10 ppm and a decoy search database option enabled. The PSM-FDR for peptides and proteins was set to 0.01. The mzid output file was used by PIT:Extract hits to extract ~160,000 peptides corresponding to Trinity generated transcript ORFs. The Trinity transcripts were mapped to the *Ae. aegypti* genome (taxid: 7159; Aedes-aegypti-Liverpool_SCAFFOLDS_AaegL3.fa, VectorBase, vectorbase.org [47] release date Apr, 2014) using GMAP [66] The output files from PIT:Extract hits and GMAP were used for PIT:Integrate, which is the core of PIT methodology, to integrate identified transcriptomic and proteomic features into a single file. In the final step of the workflow, PIT:Protein homology was used to BLAST each protein sequence against the *Ae. aegypti* (Aedes-aegypti-Liverpool_PEPTIDES_AaegL3.3, VectorBase: release date Oct, 2014)*, Cx. quinquefasciatus* (taxid: 7176; Culex-quinquefasciatus-Johannesburg_PEPTIDES_CpipJ2.2.fa, VectorBase: release date Aug, 2014), *D. melanogaster* (taxid: 7227; proteome ID UP000000803, Uniprot: release date Oct, 2014) and the NCBI non-redundant protein RefSeq databases respectively. For each protein, if the identity of the BLAST hit passed the threshold (default value is 60%) the search for that sequence stopped, if not, the sequence was BLAST searched against the NCBI non-redundant database.

MaxQuant (version 1.2.2.5) [43] in combination with the Andromeda search engine [44] were used to compare the PIT analysis with conventional proteomic analysis. The 20 .RAW files were processed and searched against a combined FASTA file containing the 6 frame translation of the *de novo* transcriptome produced by Trinity, a 6 frame translation of the official *Ae. aegypti* transcript list (Aedes-aegypti-Liverpool_TRANSCRIPTS_AaegL3.3.fa; VectorBase: release date Oct 2014) and the official *Ae. aegypti* peptide list (Aedes-aegypti-Liverpool_PEPTIDES_AaegL3.3.fa). Cysteine carbamidomethylation was set as a fixed modification and methionine oxidation and N-terminal acetylation as variable modifications in the search. Searches were performed with full tryptic digestion, a MS tolerance of 6 ppm, a maximum number of 5 modifications per peptide and a minimum peptide length of 6, a maximum of 2 missed cleavages and a maximum charge of 7. Reverse database search options were enabled and contaminants included. The MS/MS tolerance was set at 0.5 Da and the FDR for peptides and proteins was set to 0.01. A posterior error probability (PEP) score was generated for each protein. Only proteins with a PEP of less than 0.1 were considered in the analysis.

### Genome annotation

Transcripts corresponding to ‘non-*Aedes*’ insect PIT hits were searched for homology against the current *Ae. aegypti* genome assembly (Aedes-aegypti-Liverpool_SCAFFOLDS_AaegL3.fa). Alignments were downloaded in.svg format and superficially modified in Adobe Illustrator (Adobe Systems, San Jose, CA USA) for clarity only. To assess sequence quality, the genomic sequence 5,000 bases up- and down-stream of each PIT transcript alignment that corresponded with new genome annotation (*n* = 145) was downloaded, copied into Microsoft Excel (Microsoft Corporation, Redmund, WA USA) and analysed for the presence of N’s (sequencing gaps). An equal number of PIT hits corresponding to known *Ae. aegypti* genes (matched for transcript length) was similarly analysed. Supercontig chromosomal mapping data was as published [50]. The top 20% of chromosomal regions containing more than two new PIT annotations were considered ‘enriched’ for new annotation. In Fig. 2B, published transcriptomic and proteomic datasets (Additional file 6) were downloaded and transferred into Microsoft Excel. PIT hits were searched against these data based on VectorBase ID (vectorbase.org). For practical reasons proteins and transcripts lacking a Vectorbase ID were excluded from the analysis. Proteins listed in the Uniprot database (uniprot.org) were also included. All data analyses were performed in Microsoft Excel and illustrations prepared in Adobe Illustrator.

### TE analyses

The complete list of mosquito TE amino acid sequences was downloaded from tefam.biochem.vt.edu on the 31^st^ of August 2015. A complete list of TE sequences was downloaded from RepBase (RepBase21.06 on the 16^th^ of August, 2016) and compiled into a FASTA file. A combined FASTA file containing the TEfam and RepBase TEs was also generated. PIT data were searched against these sequences using the online versions of BLASTp and tBLASTn and bespoke scripts [2, 7]. Criteria for high-confidence hits were a BLAST E-value <10^−5^, query cover >45% and identity >30%, according to Pearson et al. [67]﻿ and our own empirically-determined thresholds. The top hit (according to the BLAST score) was used for all analyses. As a final quality control measure, five TE proteins identified through just one peptide were removed from the final mobilome list. The peptides associated with these five hits also matched other *Ae. aegypti* genes or TE proteins corresponding to non-related mobile elements or non-concordant ORFs in related elements. These peptides therefore do not reliably map to specific TE proteins, possibly due to poor peptide quality or because these amino acid sequences are common to many unrelated proteins. Analyses were performed in Microsoft Excel and illustrations prepared in Adobe Illustrator. Apart from in Fig. 4C, D and E, the complete set of high-confidence TEs are included in figures and tables (not just ‘non-insect’ PIT data). For Figs. 5 and 6, percent coverage of TEs in the *Ae. aegypti* genome was as published [8], copy number in the *Ae. aegypti* genome and total representation in the TEfam database were taken from tefam.biochem.vt.edu.

### Virus analyses

The ‘non-insect’ PIT data subset was searched against the GenBank database of viruses [taxid: 10239] using the online version of BLASTp (blast.ncbi.nlm.nih.gov). Only results with a good score (>50) were included; viral proteins with homology to TEs and cellular proteins were excluded. See Additional file 5 for further details.

### Statistics

Statistical significance was determined using two-tailed Student’s *t*-test for samples of equal variance in Microsoft Excel. The level of statistical significance is indicated in figure legends.

### Data statement

The transcriptomic data has been deposited to the European Nucleotide Archive (ENA) with the study accession number PRJEB13078. The mass spectrometry proteomics data has been deposited to the ProteomeXchange Consortium via the PRIDE partner repository with the dataset identifier PXD003799. The CFAV and PCLV sequences have been deposited in GenBank with the accession numbers KU936054 and KU936055, KU936056 and KU936057 respectively.

## Additional files

Additional file 1: Complete Aag2 PIT dataset. (XLSX 7329 kb)
Additional file 2: Homology of New *Ae. Aegypti* Annotation to Known Dipteran Genes. (PDF 64 kb)
Additional file 3: Non-*Aedes* Insect Genes (Pertaining to Figs. 2 and 3). List of PIT hits matching known dipteran, but not *Ae. aegypti*, genes. Supercontig and chromosomal locations, and existing gene annotation (if any), are specified. (PDF 47 kb)
Additional file 4: Complete TE Proteome Detected By PIT in Aag2 Cells. (PDF 93 kb)
Additional file 5: Additional Materials Pertaining to Virus Discovery in Aag2 Cells. Additional data (**Figure S1**) and detailed Results, Discussion, Materials and Methods, and References related to the discovery of Phasi Charoen-like virus in Aag2 cells. (PDF 312 kb)
Additional file 6: Published Proteomic and Transcriptomic Data Used in Analysis for Fig. 2B. (PDF 73 kb)
Additional file 7:
*Ae. aegypti*-Mapped PIT Hits Used in Analysis for Fig. 3A. (PDF 14 kb)

## Abbreviations

CFAV: Cell fusing agent virus
CHIKV: Chikungunya virus
DENV: Dengue virus
FBS: Foetal bovine serum
FDR: False discovery rate
gag: Group antigen
GIO: Galaxy Integrated Omics
LC-MS/MS: Liquid chromatography with coupled tandem mass spectrometry
LTR: Long-terminal repeats
MITE: Miniature inverted repeat transposable element
ORF: Open reading frame
PCLV: Phasi Charoen-like virus
PEP: Posterior error probability
PIT: Proteomics informed by transcriptomics
PLE: Penelope-like element
pol: Polymerase
PSM: Peptide-spectrum match
RNAi: RNA interference
SINE: Short interspersed element
TE: Transposable element
TIR: Terminal inverted repeat
YFV: Yellow fever virus
ZIKV: Zika virus
