# Supplementary material for: Proteomics informed by transcriptomics for characterising active transposable elements and genome annotation in Aedes aegypti
Source: BMC Genomics. 2017 Jan 19;18:101. doi: 10.1186/s12864-016-3432-5 (PMC5248466; doi:10.1186/s12864-016-3432-5)
Supplement: Additional file 2: — Homology of New Ae. Aegypti Annotation to Known Dipteran Genes. (PDF 64 kb) [file 12864_2016_3432_MOESM2_ESM.pdf]

Homology of New *Ae. Aegypti* Annotation to Known Dipteran Genes.

Trinity ID	Species*	Gene ID	Identity**	Annotation
1	*D. melanogaster*	Q9VDR1	69%	Mediator of RNA polymerase II transcription subunit 25
34	*Cx. quinquefasciatus*	CPIJ009166	73%	Short-chain dehydrogenase
45	*Cx. quinquefasciatus*	CPIJ003204	83%	DNAJ chaperone
118	*D. melanogaster*	Q9W1A9	69%	Enoki mushroom
188	*Cx. quinquefasciatus*	CPIJ013139	95%	Pre-mRNA-splicing helicase BRR2
263	*Cx. quinquefasciatus*	CPIJ008693	88%	Conserved hypothetical protein
273	*Cx. quinquefasciatus*	CPIJ006531	73%	NSFL1 cofactor p47
366	*D. melanogaster*	A1Z7T0	65%	Serine/threonine-protein kinase N
391	*Cx. quinquefasciatus*	CPIJ008099	60%	39S ribosomal protein L52, mitochondrial precursor
395	*Cx. quinquefasciatus*	CPIJ010325	69%	Conserved hypothetical protein
487	*Cx. quinquefasciatus*	CPIJ017572	63%	Focal adhesion kinase, isoform F
511	*Cx. quinquefasciatus*	CPIJ018083	97%	Conserved hypothetical protein
776	*D. melanogaster*	Q9VVJ8	87%	TORC
855	*Cx. quinquefasciatus*	CPIJ017443	84%	HP1c
876	*Cx. quinquefasciatus*	CPIJ008798	81%	Conserved hypothetical protein
930	*Cx. quinquefasciatus*	CPIJ001780	76%	Conserved hypothetical protein
1004	*Cx. quinquefasciatus*	CPIJ009801	71%	Conserved hypothetical protein
1124	*Cx. quinquefasciatus*	CPIJ011412	76%	M-phase phosphoprotein 11
1164	*Cx. quinquefasciatus*	CPIJ010476	61%	Conserved hypothetical protein
1166	*Cx. quinquefasciatus*	CPIJ000820	6%	Conserved hypothetical protein
1189	*Cx. quinquefasciatus*	CPIJ003656	72%	Luciferin 4-monooxygenase
1206	*D. melanogaster*	Q960C5	72%	CG6860, isoform A
1325	*Cx. quinquefasciatus*	CPIJ014431	63%	Conserved hypothetical protein
1407	*Cx. quinquefasciatus*	CPIJ006929	98%	Conserved hypothetical protein
1512	*Cx. quinquefasciatus*	CPIJ008057	61%	Zinc finger protein
1536	*Cx. quinquefasciatus*	CPIJ006223	97%	Plenty of SH3s, putative
1537	*Cx. quinquefasciatus*	CPIJ015803	93%	CRAG protein
1752	*Cx. quinquefasciatus*	CPIJ017756	74%	Conserved hypothetical protein
1815	*Cx. quinquefasciatus*	CPIJ007969	88%	Conserved hypothetical protein
1917	*Cx. quinquefasciatus*	CPIJ019010	81%	Protein FAM116B
1935	*Cx. quinquefasciatus*	CPIJ006912	80%	Maggie
1939	*Cx. quinquefasciatus*	CPIJ014288	84%	TAR DNA-binding protein 43
1941	*Cx. quinquefasciatus*	CPIJ005058	61%	Hypothetical protein
2016	*Cx. quinquefasciatus*	CPIJ007543	86%	Conserved hypothetical protein
2087	*Cx. quinquefasciatus*	CPIJ002507	91%	Nuclear receptor co-repressor 1
2169	*Cx. quinquefasciatus*	CPIJ009759	60%	Bub1, putative
2196	*D. melanogaster*	P91632	87%	Mushroom-body expressed, isoform A
2272	*Cx. quinquefasciatus*	CPIJ000576	68%	Sulfotransferase
2360	*Cx. quinquefasciatus*	CPIJ004120	62%	Huntingtin
2376	*Cx. quinquefasciatus*	CPIJ000180	88%	Conserved hypothetical protein
2391	*Cx. quinquefasciatus*	CPIJ002335	73%	ATP-dependent RNA helicase DDX51
2548	*D. melanogaster*	Q9VYR2	61%	CG1492
2560	*D. melanogaster*	Q9VL63	85%	UPF0430 protein CG31712
2561	*Cx. quinquefasciatus*	CPIJ003815	78%	Conserved hypothetical protein
2599	*Cx. quinquefasciatus*	CPIJ005301	91%	p19 protein, putative
2799	*Cx. quinquefasciatus*	CPIJ006174	85%	Down syndrome cell adhesion molecule, putative
2903	*Cx. quinquefasciatus*	CPIJ008210	64%	Conserved hypothetical protein

Homology of New *Ae. Aegypti* Annotation to Known Dipteran Genes. (continued)

Trinity ID	Species*	Gene ID	Identity**	Annotation
2949	*Cx. quinquefasciatus*	CPIJ018512	60%	Cell division cycle and apoptosis regulator protein 1
3014	*Cx. quinquefasciatus*	CPIJ003976	86%	Gar2, putative
3084	*Cx. quinquefasciatus*	CPIJ007479	87%	Conserved hypothetical protein
3089	*Cx. quinquefasciatus*	CPIJ013564	93%	Conserved hypothetical protein
3140	*Cx. quinquefasciatus*	CPIJ007395	81%	Conserved hypothetical protein
3166	*Cx. quinquefasciatus*	CPIJ002759	86%	Dedicator of cytokinesis protein 1
3168	*Cx. quinquefasciatus*	CPIJ000382	69%	Conserved hypothetical protein
3214	*Cx. quinquefasciatus*	CPIJ012115	71%	Conserved hypothetical protein
3233	*Cx. quinquefasciatus*	CPIJ014388	80%	Conserved hypothetical protein
3234	*Cx. quinquefasciatus*	CPIJ016881	78%	Conserved hypothetical protein
3338	*Cx. quinquefasciatus*	CPIJ016254	99%	Ubiquitin domain-containing protein 1
3434	*Cx. quinquefasciatus*	CPIJ007342	61%	Conserved hypothetical protein
3462	*Cx. quinquefasciatus*	CPIJ012976	100%	Conserved hypothetical protein
3504	*Cx. quinquefasciatus*	CPIJ017247	98%	Microsomal signal peptidase 18 kDa subunit
3521	*Cx. quinquefasciatus*	CPIJ004184	72%	DNA-repair protein complementing XP-G cells
3673	*D. melanogaster*	Q24523	60%	Protein bunched, class 2/F/G isoform
3702	*Cx. quinquefasciatus*	CPIJ015923	78%	Conserved hypothetical protein
3911	*Cx. quinquefasciatus*	CPIJ010852	82%	Conserved hypothetical protein
3915	*Cx. quinquefasciatus*	CPIJ010568	61%	Aromatic amino acid decarboxylase
3951	*Cx. quinquefasciatus*	CPIJ002564	64%	Ubiquitin-specific protease
4063	*Cx. quinquefasciatus*	CPIJ005294	89%	Collagen IV alpha 1 chain precursor
4068	*Cx. quinquefasciatus*	CPIJ018234	93%	Translocon-associated protein subunit alpha precursor
4179	*D. melanogaster*	P25822	66%	Maternal protein pumilio
4182	*Cx. quinquefasciatus*	CPIJ009487	63%	Conserved hypothetical protein
4622	*Cx. quinquefasciatus*	CPIJ000297	77%	5,10-methylenetetrahydrofolate reductase, putative
4654	*Cx. quinquefasciatus*	CPIJ001645	75%	Conserved hypothetical protein
4656	*Cx. quinquefasciatus*	CPIJ001569	68%	Conserved hypothetical protein
4755	*Cx. quinquefasciatus*	CPIJ019325	98%	Conserved hypothetical protein
4822	*Cx. quinquefasciatus*	CPIJ018144	62%	Zinc finger protein 358
4849	*Cx. quinquefasciatus*	CPIJ005295	76%	Collagen alpha-2 (IV) chain precursor
4853	*Cx. quinquefasciatus*	CPIJ013032	96%	Dystrophin major muscle isoform
5019	*Cx. quinquefasciatus*	CPIJ003129	74%	Conserved hypothetical protein
5099	*Cx. quinquefasciatus*	CPIJ010406	88%	Light protein
5241	*Cx. quinquefasciatus*	CPIJ008209	63%	Conserved hypothetical protein
5272	*Cx. quinquefasciatus*	CPIJ015585	68%	Arf6 guanine nucleotide exchange factor
5307	*Cx. quinquefasciatus*	CPIJ012464	90%	Conserved hypothetical protein
5467	*Cx. quinquefasciatus*	CPIJ012359	74%	Conserved hypothetical protein
5469	*Cx. quinquefasciatus*	CPIJ002999	100%	Conserved hypothetical protein
5498	*Cx. quinquefasciatus*	CPIJ008314	90%	Sideroflexin 1,2,3
5623	*Cx. quinquefasciatus*	CPIJ013695	90%	Bub1, putative
5634	*Cx. quinquefasciatus*	CPIJ006272	92%	Conserved hypothetical protein
5720	*D. melanogaster*	Q9VDU9	66%	Histamine-gated chloride channel alpha1 subunit
5870	*Cx. quinquefasciatus*	CPIJ007864	80%	Conserved hypothetical protein
5872	*Cx. quinquefasciatus*	CPIJ005885	96%	XPA-binding protein 2
5904	*Cx. quinquefasciatus*	CPIJ005652	98%	40S ribosomal protein S27
5975	*Cx. quinquefasciatus*	CPIJ003271	94%	Broad complex isoform Z3
6069	*Cx. quinquefasciatus*	CPIJ002505	82%	Conserved hypothetical protein

Homology of New *Ae. Aegypti* Annotation to Known Dipteran Genes. (continued)

Trinity ID	Species*	Gene ID	Identity**	Annotation
6097	*Cx. quinquefasciatus*	CPIJ002709	70%	Conserved hypothetical protein
6137	*Cx. quinquefasciatus*	CPIJ001922	87%	Dedicator of cytokinesis protein 9
6176	*Cx. quinquefasciatus*	CPIJ001165	75%	Ubiquitin conjugation factor E4 A
6221	*Cx. quinquefasciatus*	CPIJ006398	91%	Conserved hypothetical protein
6234	*Cx. quinquefasciatus*	CPIJ018588	79%	Conserved hypothetical protein
6254	*Cx. quinquefasciatus*	CPIJ015253	63%	Zinc carboxypeptidase A 1 precursor
6272	*Cx. quinquefasciatus*	CPIJ009584	67%	Fibrinogen and fibronectin, putative
6289	*Cx. quinquefasciatus*	CPIJ017497	61%	Transcriptional activator HAP2
6296	*Cx. quinquefasciatus*	CPIJ002228	71%	Sentrin/sumo-specific protease (Senp7)
6311	*D. melanogaster*	Q9W1R3	76%	Golgin-245 orthologue
6339	*Cx. quinquefasciatus*	CPIJ012266	68%	DNA polymerase subunit gamma 1, mitochondrial precursor
6359	*Cx. quinquefasciatus*	CPIJ009979	70%	Eukaryotic translation initiation factor 4 gamma 3
6383	*Cx. quinquefasciatus*	CPIJ018110	67%	Conserved hypothetical protein
6388	*Cx. quinquefasciatus*	CPIJ012977	73%	Pcnx protein
6501	*Cx. quinquefasciatus*	CPIJ001647	64%	Predicted protein
6594	*Cx. quinquefasciatus*	CPIJ006574	96%	Conserved hypothetical protein
6619	*Cx. quinquefasciatus*	CPIJ001572	83%	Hypothetical protein
6635	*Cx. quinquefasciatus*	CPIJ011849	64%	Adenosine deaminase acting on RNA
7008	*Cx. quinquefasciatus*	CPIJ000671	78%	Caspase precursor
7032	*Cx. quinquefasciatus*	CPIJ017118	61%	Conserved hypothetical protein
7125	*Cx. quinquefasciatus*	CPIJ010284	85%	Beta-lactamase hcpA precursor
7131	*Cx. quinquefasciatus*	CPIJ005253	88%	Conserved hypothetical protein
7135	*Cx. quinquefasciatus*	CPIJ002341	78%	Peroxisomal targeting signal 1 receptor
7147	*Cx. quinquefasciatus*	CPIJ017711	84%	Hypothetical protein
7179	*Cx. quinquefasciatus*	CPIJ008394	61%	PHD finger protein 10
7184	*Cx. quinquefasciatus*	CPIJ002077	75%	Aspartyl/asparaginyl beta-hydroxylase
7263	*Cx. quinquefasciatus*	CPIJ013494	99%	Enhancer of rudimentary homolog
7299	*D. melanogaster*	A1Z7T2	65%	Protein kinase related to protein kinase N, isoform F
7528	*Cx. quinquefasciatus*	CPIJ007378	79%	Conserved hypothetical protein
7783	*Cx. quinquefasciatus*	CPIJ017651	87%	Conserved hypothetical protein
7791	*Cx. quinquefasciatus*	CPIJ012523	95%	Coatomer subunit gamma
7813	*Cx. quinquefasciatus*	CPIJ014689	93%	Transcriptional repressor protein YY1
7819	*Cx. quinquefasciatus*	CPIJ011546	65%	Protease m1 zinc metalloprotease
7835	*Cx. quinquefasciatus*	CPIJ000059	84%	Conserved hypothetical protein
7941	*Cx. quinquefasciatus*	CPIJ009160	84%	Sorting nexin
8033	*Cx. quinquefasciatus*	CPIJ010004	64%	DNA topoisomerase 2-binding protein 1
8089	*Cx. quinquefasciatus*	CPIJ003562	71%	Conserved hypothetical protein
8094	*Cx. quinquefasciatus*	CPIJ018589	69%	Conserved hypothetical protein
8096	*Cx. quinquefasciatus*	CPIJ007709	91%	Afadin
8143	*D. melanogaster*	Q9VXU2	66%	Golgi microtubule-associated protein, isoform A
8326	*Cx. quinquefasciatus*	CPIJ009666	85%	Conserved hypothetical protein
8683	*Cx. quinquefasciatus*	CPIJ006993	71%	Protein-lysine 6-oxidase, putative
8697	*Cx. quinquefasciatus*	CPIJ003321	89%	Tetracycline resistance
8718	*Cx. quinquefasciatus*	CPIJ001315	60%	K10 protein
8865	*Cx. quinquefasciatus*	CPIJ012238	73%	Protein phosphatase 4
8871	*Cx. quinquefasciatus*	CPIJ001086	83%	Conserved hypothetical protein
9039	*Cx. quinquefasciatus*	CPIJ019093	79%	Conserved hypothetical protein

Homology of New *Ae. Aegypti* Annotation to Known Dipteran Genes. (continued)

Trinity ID	Species*	Gene ID	Identity**	Annotation
9058	*D. melanogaster*	P17133	96%	U1 small nuclear ribonucleoprotein 70 kDa
9337	*Cx. quinquefasciatus*	CPIJ000280	95%	Conserved hypothetical protein
9419	*Cx. quinquefasciatus*	CPIJ002893	76%	Conserved hypothetical protein
9431	*Cx. quinquefasciatus*	CPIJ003133	77%	Protein phosphatases pp1 regulatory subunit

* Dipteran species (*D. melanogaster* or *Cx. quinquefasciatus*) in which closest gene match was identified.

** Amino acid identity.

Only top BLAST hits shown. Gene ID is Vectorbase ID (*Cx. quinquefasciatus*) or Uniprot ID (*D. melanogaster*).
