# Supplementary material for: Proteomics informed by transcriptomics for characterising active transposable elements and genome annotation in Aedes aegypti
Source: BMC Genomics. 2017 Jan 19;18:101. doi: 10.1186/s12864-016-3432-5 (PMC5248466; doi:10.1186/s12864-016-3432-5)
Supplement: Additional file 3: — Non-Aedes Insect Genes (Pertaining to Figs. 2 and 3). List of PIT hits matching known dipteran, but not Ae. aegypti, genes. Supercontig and chromosomal locations, and existing gene annotation (if any), are specified. (PDF 47 kb) [file 12864_2016_3432_MOESM3_ESM.pdf]

Non-*Aedes* Insect Genes (Pertaining to Fig. 2 and 3).
If not specified, supercontig has not been assigned chromosomal location.

	Trinity ID	Supercontig	Chromosomal Location	Annotation
Homology to Known *Ae. aegypti* Genes	75	1.1657		AAEL012117
	545	1.93		AAEL003613
	1476	1.9		AAEL000509
	3324	1.1411		AAEL015034
	3738	1.195		AAEL006222
	4203	1.4383		AAEL017530
	4627	1.82	1q43	AAEL801258
	5546	1.1647		AAEL015242
	6150	1.11	3q11	AAEL000565
	6235	1.327		AAEL008459
	6462	1.333		AAEL008586
	7068	1.31	2p13	AAEL001388
	7478	1.542		AAEL011111
	7738	1.8	3p12	AAEL000429
	8084	1.588		AAEL011533
	8390	1.481		AAEL010501
	8565	1.507		AAEL010781
	8804	1.321		AAEL008382
	8886	1.55	2q24	AAEL002382
	9220	1.465		AAEL017054
	9268	1.716		AAEL012608
New Annotation	1	1.931		
	34	1.956		
	45	1.402		
	118	1.541		
	188	1.1224		
	263	1.68	1q44	
	273	1.1096		
	366	1.36	2p42	
	391	1.4	1p34	
	395	1.1117		
	487	1.126		
	511	1.35		
	776	1.231		
	855	1.97		
	876	1.3	3q12	
	930	1.154		
	1004	1.148	1p34	
	1124	1.1159		
	1164	1.191		
	1166	1.443		
	1189	1.229		
	1206	1.575		
	1325	1.17	3p22	

Non-*Aedes* Insect Genes (Pertaining to Fig. 2 and 3). (continued)

	Trinity ID	Supercontig	Chromosomal Location	Annotation
New Annotation	1407	1.735		
	1512	1.567		
	1536	1.16	3q21	
	1537	1.754		
	1752	1.787		
	1815	1.253		
	1917	1.833		
	1935	1.704	2p34	
	1939	1.389		
	1941	1.326	1q12	
	2016	1.132	2p32	
	2087	1.418		
	2169	1.171	3q11	
	2196	1.28	2p23	
	2272	1.619		
	2360	1.147	1q13	
	2376	1.225		
	2391	1.54		
	2548	1.160	3q21	
	2560	1.11	3q11	
	2561	1.271		
	2599	1.964		
	2799	1.490		
	2903	1.386	3p42	
	2949	1.753		
	3014	1.856		
	3084	1.8	3p12	
	3089	1.13	3q44	
	3140	1.50	1p33	
	3166	1.1628		
	3168	1.396		
	3214	1.46	2p21	
	3233	1.185		
	3234	1.474		
	3338	1.65		
	3434	1.219		
	3462	1.369		
	3504	1.1104		
	3521	1.204		
	3673	1.274	2q12	
	3702	1.213		
	3911	1.199		
	3915	1.1002		
	3951	1.956		
	4063	1.291		
	4068	1.81	1q21	

Non-*Aedes* Insect Genes (Pertaining to Fig. 2 and 3). (continued)

	Trinity ID	Supercontig	Chromosomal Location	Annotation
New Annotation	4179	1.694		
	4182	1.715		
	4622	1.2330		
	4654	1.488		
	4656	1.352	2q11	
	4755	1.510		
	4822	1.185		
	4849	1.291		
	4853	1.215		
	5019	1.80		
	5099	1.24	2q24	
	5241	1.386	3p42	
	5272	1.111	1p21	
	5307	1.75	1p14	
	5467	1.283		
	5469	1.559		
	5498	1.1152		
	5623	1.184		
	5634	1.274	2q12	
	5720	1.236		
	5870	1.52	2p42	
	5872	1.18		
	5904	1.495		
	5975	1.324		
	6069	1.418		
	6097	1.125		
	6137	1.128	2q33	
	6176	1.777	1p33	
	6221	1.161	1p33	
	6234	1.191		
	6254	1.1691		
	6272	1.174		
	6289	1.293		
	6296	1.31	2p13	
	6311	1.442		
	6339	1.1171		
	6359	1.23	3p44	
	6383	1.384	1p32	
	6388	1.369		
	6501	1.488		
	6594	1.332	2p23	
	6619	1.352	2q11	
	6635	1.59	1p31	
	7008	1.791		
	7032	1.9		
	7125	1.73	1q41	

Non-*Aedes* Insect Genes (Pertaining to Fig. 2 and 3). (continued)

	Trinity ID	Supercontig	Chromosomal Location	Annotation
New Annotation	7131	1.201		
	7135	1.497		
	7147	1.307		
	7179	1.393		
	7184	1.341	2p44	
	7263	1.571		
	7299	1.36	2p42	
	7528	1.20	2p44	
	7783	1.1510		
	7791	1.244		
	7813	1.481		
	7819	1.656		
	7835	1.55	2q24	
	7941	1.251		
	8033	1.450		
	8089	1.191		
	8094	1.191		
	8096	1.117	1q33	
	8143	1.603		
	8326	1.542		
	8683	1.190		
	8697	1.123	1p12	
	8718	1.180		
	8865	1.268		
	8871	1.82	1q43	
	9039	1.932		
	9058	1.570		
	9337	1.210		
	9419	1.240	2q24	
	9431	1.80		
Ambiguous	1792			
	4353			
	6114			
	6489			
	6783			
	7728			
	7730			
	8587			
	8958			
	9053			
	9256			
No Match	909			
	1051			
	1589			
	2357			
	2623			

Non-*Aedes* Insect Genes (Pertaining to Fig. 2 and 3). (continued)

	Trinity ID	Supercontig	Chromosomal Location	Annotation
No Match	2727			
	2974			
	2995			
	3026			
	3093			
	3098			
	3210			
	3392			
	3427			
	3846			
	3852			
	4288			
	4479			
	4862			
	4878			
	4957			
	5502			
	6146			
	6464			
	6478			
	6902			
	7255			
	7354			
	7441			
	7737			
	7801			
	7893			
	7912			
	8485			
	9050			
	9227			
	9273			
	9393			
