# Supplementary material for: Proteomics informed by transcriptomics for characterising active transposable elements and genome annotation in Aedes aegypti
Source: BMC Genomics. 2017 Jan 19;18:101. doi: 10.1186/s12864-016-3432-5 (PMC5248466; doi:10.1186/s12864-016-3432-5)
Supplement: Additional file 4: — Complete TE Proteome Detected By PIT in Aag2 Cells. (PDF 93 kb) [file 12864_2016_3432_MOESM4_ESM.pdf]

Complete TE Proteome Detected By PIT in Aag2 Cells.

Class	Order/Subclass	Clade/Superfamily	Element	ORF	TEfam ID	Mosquito*	PIT AA Length	Query Cover	Identity	Trinity ID
Class I	LTR	BEL	Ele1	gag	TF000042	*Ae. aegypti*	811	99%	48%	402
			Ele1	gag	TF000042	*Ae. aegypti*	1269	76%	42%	955
			Ele1	gag	TF000042	*Ae. aegypti*	1933	84%	44%	956
			Ele10	gag	TF000092	*Ae. aegypti*	815	99%	44%	2271
			**Ele10**	**gag**	**TF000092**	***Ae. aegypti***	**1168**	**99%**	**33%**	**3264**
			Ele10	gag	TF000092	*Ae. aegypti*	949	99%	48%	4868
			Ele10	gag	TF000092	*Ae. aegypti*	767	99%	47%	4869
			Ele45	gag	TF000244	*Ae. aegypti*	69	89%	68%	6477
			Ele77	pol	TF000260	*Ae. aegypti*	104	99%	85%	9231
			Ele97	gag	TF000270	*Ae. aegypti*	161	100%	90%	4488
			Ele23	gag	TF000278	*Ae. aegypti*	336	99%	83%	3203
			Ele121	gag	TF000282	*Ae. aegypti*	812	71%	65%	7201
			Ele147	gag	TF000295	*Ae. aegypti*	444	99%	72%	5203
			Ele147	gag	TF000295	*Ae. aegypti*	207	100%	76%	9023
			Ele153	gag	TF000298	*Ae. aegypti*	83	100%	100%	8309
			**Ele181**	**gag**	**TF000313**	***Ae. aegypti***	**104**	**100%**	**64%**	**3381**
			Ele24	gag	TF000326	*Ae. aegypti*	177	100%	69%	4222
			Ele26	gag	TF000327	*Ae. aegypti*	357	97%	72%	3396
			Ele34	gag	TF000332	*Ae. aegypti*	139	99%	80%	4720
			Ele227	gag	TF000507	*Ae. aegypti*	73	100%	100%	9160
			Ele84	gag	TF000514	*Ae. aegypti*	196	100%	60%	5873

Complete TE Proteome Detected By PIT in Aag2 Cells. (continued)

Class	Order/Subclass	Clade/Superfamily	Element	ORF	TEfam ID	Mosquito*	PIT AA Length	Query Cover	Identity	Trinity ID
Class I	LTR	BEL	Ele86	gag	TF000515	*Ae. aegypti*	88	100%	53%	5754
			Ele90	gag	TF000517	*Ae. aegypti*	572	99%	48%	5158
			Ele226	gag	TF000777	*Ae. aegypti*	267	100%	87%	8358
			Ele220	gag	TF000788	*Ae. aegypti*	83	93%	38%	1400
			Ele204	gag	TF000800	*Ae. aegypti*	80	93%	57%	6338
			Ele24	gag	TF003410	*An. gambiae*	286	97%	58%	5038
			**Ele24**	**gag**	**TF001456**	***Cx. quinquefasciatus***	**435**	**95%**	**62%**	**5543**
			Ele24	gag	TF001456	*Cx. quinquefasciatus*	154	95%	48%	6976
			Ele24	gag	TF001456	*Cx. quinquefasciatus*	388	100%	74%	7002
			**Ele23**	**gag**	**TF001470**	***Cx. quinquefasciatus***	**778**	**95%**	**43%**	**6977**
			Ele51	gag	TF001550	*Cx. quinquefasciatus*	116	82%	38%	4423
			Ele68	gag	TF001567	*Cx. quinquefasciatus*	202	99%	65%	6696
			Ele68	gag	TF001567	*Cx. quinquefasciatus*	149	100%	77%	7991
		Ty1/copia	**Ele1**	**pol**	**TF000130**	***Ae. aegypti***	**1176**	**81%**	**42%**	**2155**
			Ele39	gag	TF000661	*Ae. aegypti*	689	94%	99%	3822
			Ele188	gag	TF001077	*Ae. aegypti*	774	69%	62%	6729
			Ele2	gag	TF003443	*An. gambiae*	88	100%	69%	6307
			Ele4	gag	TF003445	*An. gambiae*	108	76%	75%	4632
			Ele8	gag	TF003449	*An. gambiae*	139	94%	70%	5994
			Ele12	gag	TF003453	*An. gambiae*	115	100%	63%	192
			Ele12	gag	TF003453	*An. gambiae*	249	97%	73%	2237

Complete TE Proteome Detected By PIT in Aag2 Cells. (continued)

Class	Order/Subclass	Clade/Superfamily	Element	ORF	TEfam ID	Mosquito*	PIT AA Length	Query Cover	Identity	Trinity ID
Class I	LTR	Ty1/copia	Ele8	gag	TF001523	*Cx. quinquefasciatus*	155	94%	53%	4022
			Ele9	gag	TF001524	*Cx. quinquefasciatus*	110	92%	82%	2080
			Ele9	gag	TF001524	*Cx. quinquefasciatus*	114	57%	41%	5800
			Ele9	gag	TF001524	*Cx. quinquefasciatus*	121	99%	63%	8491
			Ele14	gag	TF001529	*Cx. quinquefasciatus*	118	98%	59%	6400
			Ele17	gag	TF001581	*Cx. quinquefasciatus*	162	85%	65%	4571
			Ele18	gag	TF001582	*Cx. quinquefasciatus*	526	99%	47%	4431
			**Ele19**	**gag**	**TF001583**	***Cx. quinquefasciatus***	**276**	**85%**	**43%**	**3370**
			Ele26	gag	TF001590	*Cx. quinquefasciatus*	444	96%	61%	298
			Ele32	gag	TF001594	*Cx. quinquefasciatus*	195	82%	72%	6132
			Ele32	gag	TF001594	*Cx. quinquefasciatus*	122	71%	69%	6133
			Ele34	gag	TF001596	*Cx. quinquefasciatus*	294	99%	55%	8976
		Ty3/gypsy	Ele7	pol	TF000099	*Ae. aegypti*	134	100%	99%	8866
			Ele19	gag	TF000110	*Ae. aegypti*	125	100%	100%	2793
			Ele26	gag	TF000117	*Ae. aegypti*	206	92%	98%	4178
			**Ele26**	**gag**	**TF000117**	***Ae. aegypti***	**1428**	**74%**	**35%**	**6210**
			**Ele26**	**gag**	**TF000117**	***Ae. aegypti***	**1298**	**93%**	**35%**	**8288**
			**Ele28**	**gag**	**TF000119**	***Ae. aegypti***	**115**	**84%**	**39%**	**1383**
			**Ele28**	**gag**	**TF000119**	***Ae. aegypti***	**174**	**89%**	**43%**	**452**
			Ele29	gag	TF000120	*Ae. aegypti*	350	89%	46%	7939
			Ele30	pol	TF000121	*Ae. aegypti*	497	99%	59%	2218

Complete TE Proteome Detected By PIT in Aag2 Cells. (continued)

Class	Order/Subclass	Clade/Superfamily	Element	ORF	TEfam ID	Mosquito*	PIT AA Length	Query Cover	Identity	Trinity ID
Class I	LTR	Ty3/gypsy	Ele32	gag	TF000123	*Ae. aegypti*	202	94%	77%	3616
			Ele33	pol	TF000124	*Ae. aegypti*	199	96%	69%	4176
			**Ele40**	**gag**	**TF000135**	***Ae. aegypti***	**338**	**100%**	**100%**	**5565**
			Ele43	pol	TF000138	*Ae. aegypti*	86	86%	65%	2550
			Ele50	'env'	TF000145	*Ae. aegypti*	242	100%	97%	4625
			Ele50	'env'	TF000145	*Ae. aegypti*	137	100%	85%	4626
			Ele54	'env'	TF000317	*Ae. aegypti*	533	100%	99%	7261
			Ele55	'env'	TF000318	*Ae. aegypti*	250	100%	99%	2813
			Ele65	gag	TF000337	*Ae. aegypti*	463	100%	53%	6239
			Ele68	gag	TF000340	*Ae. aegypti*	409	99%	66%	4711
			Ele69	gag	TF000341	*Ae. aegypti*	468	97%	98%	3730
			Ele83	gag	TF000368	*Ae. aegypti*	85	98%	54%	4772
			Ele84	'env'	TF000369	*Ae. aegypti*	192	80%	62%	4205
			**Ele97**	**gag**	**TF000379**	***Ae. aegypti***	**751**	**99%**	**44%**	**2546**
			Ele98	pol	TF000380	*Ae. aegypti*	205	98%	57%	6938
			Ele101	gag	TF000382	*Ae. aegypti*	80	100%	96%	5479
			Ele104	pol	TF000385	*Ae. aegypti*	492	100%	99%	5488
			Ele107	gag	TF000388	*Ae. aegypti*	212	99%	46%	8265
			Ele113	gag	TF000413	*Ae. aegypti*	73	100%	89%	5360
			Ele120	gag	TF000417	*Ae. aegypti*	78	83%	45%	6629
			Ele121	'env'	TF000418	*Ae. aegypti*	107	80%	48%	5210

Complete TE Proteome Detected By PIT in Aag2 Cells. (continued)

Class	Order/Subclass	Clade/Superfamily	Element	ORF	TEfam ID	Mosquito*	PIT AA Length	Query Cover	Identity	Trinity ID
Class I	LTR	Ty3/gypsy	Ele122	'env'	TF000419	*Ae. aegypti*	102	100%	100%	8311
			Ele154	gag	TF000540	*Ae. aegypti*	282	100%	99%	656
			Ele156	gag	TF000542	*Ae. aegypti*	310	99%	55%	8637
			Ele158	pol	TF000544	*Ae. aegypti*	141	100%	77%	7310
			Ele158	pol	TF000544	*Ae. aegypti*	237	100%	80%	7311
			Ele190	gag	TF000932	*Ae. aegypti*	287	78%	67%	2732
			Ele183	pol	TF000936	*Ae. aegypti*	98	97%	59%	2091
			Ele178	pol	TF000940	*Ae. aegypti*	110	100%	66%	2436
			Ele10	pol	TF002594	*An. arabiensis*	290	94%	50%	6559
			Ele50	gag	TF003509	*An. gambiae*	138	55%	57%	9384
			**Ele6**	**gag**	**TF002676**	***An. stephensi***	**728**	**100%**	**57%**	**724**
			Ele20	gag	TF001499	*Cx. quinquefasciatus*	451	93%	43%	8125
			**Ele22**	**gag**	**TF001500**	***Cx. quinquefasciatus***	**359**	**65%**	**35%**	**2678**
			Ele22	gag	TF001500	*Cx. quinquefasciatus*	87	100%	64%	9423
			Ele23	gag	TF001503	*Cx. quinquefasciatus*	129	93%	71%	8790
			**Ele40**	**gag**	**TF001597**	***Cx. quinquefasciatus***	**677**	**100%**	**49%**	**1630**
			Ele59	gag	TF001606	*Cx. quinquefasciatus*	69	60%	43%	5794
			Ele50	pol	TF001609	*Cx. quinquefasciatus*	131	100%	74%	3989
	Non-LTR	CR1	Ele18	ORF2	TF002960	*An. dirus*	104	94%	39%	3859
			Ele6	ORF2	TF002797	*An. epiroticus*	114	95%	35%	3922
			Ele3	ORF1	TF001345	*An. gambiae*	193	89%	32%	1306

Complete TE Proteome Detected By PIT in Aag2 Cells. (continued)

Class	Order/Subclass	Clade/Superfamily	Element	ORF	TEfam ID	Mosquito*	PIT AA Length	Query Cover	Identity	Trinity ID
Class I	Non-LTR	CR1		ORF2	TF001349	*An. gambiae*	579	98%	47%	5304
			Ele1	ORF2	TF003184	*An. merus*	77	88%	53%	4275
			Ele16	ORF2	TF003213	*An. merus*	137	99%	47%	4445
			Ele24	ORF2	TF003221	*An. merus*	561	95%	38%	3559
		I factor	Ele1	ORF2	TF000020	*Ae. aegypti*	116	100%	60%	2555
			Ele1	ORF2	TF000020	*Ae. aegypti*	188	96%	30%	3042
			Ele1	ORF2	TF000020	*Ae. aegypti*	213	97%	30%	4861
		Jockey	Ele1	ORF2	TF000019	*Ae. aegypti*	450	100%	99%	4096
			Ele3	ORF2	TF000069	*Ae. aegypti*	78	91%	54%	1382
			Ele3	ORF2	TF000069	*Ae. aegypti*	303	98%	57%	8938
		L1	Ele5	ORF1	TF000086	*An. gambiae*	149	82%	42%	2159
			Ele2	ORF2	TF001353	*An. gambiae*	134	94%	52%	908
			Ele4	ORF2	TF003258	*An. merus*	223	99%	38%	4791
		LOA	Ele1	ORF1	TF000021	*Ae. aegypti*	196	97%	39%	5533
			Ele1	ORF1	TF000021	*Ae. aegypti*	252	99%	35%	6238
		Loner	Ele2	ORF2	TF003111	*An. funestus*	78	98%	62%	7445
			Ele3	ORF2	TF003112	*An. funestus*	82	90%	47%	2728
		Outcast	Ele5	ORF2	TF002548	*An. arabiensis*	146	69%	39%	4721
			Ele6	ORF2	TF000083	*An. gambiae*	126	84%	38%	4873
			Ele4	ORF2	TF003267	*An. merus*	294	98%	44%	314
		R1	Ele5	ORF2	TF002830	*An. epiroticus*	114	76%	46%	1402

Complete TE Proteome Detected By PIT in Aag2 Cells. (continued)

Class	Order/Subclass	Clade/Superfamily	Element	ORF	TEfam ID	Mosquito*	PIT AA Length	Query Cover	Identity	Trinity ID
Class I	Non-LTR	R4	Ele1	ORF1	TF000040	*Ae. aegypti*	82	95%	99%	6342
		RTE	Ele3	ORF2	TF002839	*An. epiroticus*	97	100%	59%	4841
			Ele2	ORF1	TF001352	*An. gambiae*	63	53%	38%	9463
Class II	Cut and Paste	hAT	Ele8	ORF1	TF001635	*Cx. quinquefasciatus*	234	96%	37%	6151
		Mutator	1.1	ORF1	TF001628	*Cx. quinquefasciatus*	398	92%	32%	4663
		Tc1-Mariner	**Ele3**	**ORF1**	**TF001534**	***Cx. quinquefasciatus***	**159**	**93%**	**33%**	**5639**
			Ele3	ORF1	TF001534	*Cx. quinquefasciatus*	82	100%	78%	5750
			**Ele7**	**ORF1**	**TF001538**	***Cx. quinquefasciatus***	**195**	**52%**	**31%**	**3084**
			Ele7	ORF1	TF001538	*Cx. quinquefasciatus*	79	91%	40%	6452
			Ele7	ORF1	TF001538	*Cx. quinquefasciatus*	87	95%	54%	6680
			Ele1	ORF1	TF001539	*Cx. quinquefasciatus*	207	99%	73%	6526

* Mosquito genome in which TEfam element was originally described.

Bold typeface indicates Trinity transcripts associated with two or more peptides.
