# Supplementary material for: Proteomics informed by transcriptomics for characterising active transposable elements and genome annotation in Aedes aegypti
Source: BMC Genomics. 2017 Jan 19;18:101. doi: 10.1186/s12864-016-3432-5 (PMC5248466; doi:10.1186/s12864-016-3432-5)
Supplement: Additional file 5: — Additional Materials Pertaining to Virus Discovery in Aag2 Cells. Additional data (Figure S1) and detailed Results, Discussion, Materials and Methods, and References related to the discovery of Phasi Charoen-like virus in Aag2 cells. (PDF 312 kb) [file 12864_2016_3432_MOESM5_ESM.pdf]

ADDITIONAL MATERIALS PERTAINING TO VIRUS DISCOVERY IN AAG2 CELLS

Results

Aag2 cells are known to be persistently infected with the flavivirus cell fusing agent virus (CFAV) [1]. We thus reasoned that some of our non-mosquito hits may be virus-derived, and performed a protein BLAST search against all viruses in GenBank. We confirmed the presence of CFAV proteins (Fig. S1A), and aligned our raw RNA-seq reads to the CFAV genome to confirm complete coverage at the RNA level (Fig. S1B). The prominent peak across the 3' untranslated region (UTR) (Fig. S1B) corresponds to the subgenomic flavivirus RNA (sfRNA) that accumulates in infected cells [2]. The consensus sequence of our virus is virtually identical to the published sequence of the CFAV isolate infecting Aag2 cells (99% protein and nucleotide homology) [3].

We also detected peptides matching the insect-specific bunyavirus Phasi Charoen-like virus (PCLV) (Fig. S1A). Bunyaviruses are negative-sense RNA viruses encoding three genome segments [4], and we detected proteins encoded by the large (L) and medium (M) segments (Fig. S1A). We had almost complete RNA-seq coverage across these two segments (Fig. S1Ci and ii). The low coverage across the small (S) segment (Fig. S1Ciii) likely prohibited PIT transcript assembly, explaining why no S-derived peptides were detected (Fig. S1A). Bunyavirus RNA does not accumulate in equimolar ratios in infected cells [5, 6], and the RNA-seq data suggested that L is the most, and S the least, abundant PCLV RNA in Aag2 cells (Fig. S1C). It remained possible that these RNAs and peptides are not derived from a full-length replicating virus. Using a strand-specific PCR targeting the extreme ends of each segment, we detected negative-sense genomic viral RNA (vRNA) and positive-sense complementary RNA (cRNA) of the expected size for all segments (Fig. S1D). Since cRNA synthesis is essential for bunyavirus replication [4], these data confirm the presence of actively replicating, full-length PCLV. We sequenced these PCR amplicons to fill gaps in our RNA-seq coverage (Fig. S1C). The consensus Aag2 PCLV sequence shares 95-99% homology at the nucleotide level, and 97-99% at the amino acid level, with environmental isolates across all three genome segments [7, 8].

It is of note that we would not have identified PCLV in our sample had we restricted our analysis to proteomic hits identified by two or more peptides. Our molecular data nevertheless demonstrate that PCLV is a *bona fide* replicating virus infecting Aag2 cells, and show that PIT hits with just one peptide match can provide biologically relevant information. Our data confirm the utility of PIT in viral discovery, and identify PCLV as a previously unknown contaminant of the commonly used Aag2 cell line.

Discussion

PIT has been developed and used to identify transcripts and proteins from multiple genomes including cells/organisms with infectious agents [9, 10]. Here, we identified PCLV as an unrecognised contaminant of Aag2 cells. We detected all three full-length negative-sense genome and positive-sense antigenome segments, demonstrating active viral replication. We found PCLV in two Aag2 clones from separate labs, while ATC-10 (*Ae. aegypti*) cells, the *Ae. albopictus* cell lines ATC-15, C6/36, C7/10 and RML-12, and S2 (*D. melanogaster*) cells were all negative for PCLV (data not shown). At least 20% of wild *Ae. aegypti* females harbour PCLV [11], and since Aag2 cells were generated from a number of embryos [12, 13], it seems likely that PCLV has been present since the establishment of this cell line. Aag2 cells are perhaps the most commonly used *Ae. aegypti* cell line, and the presence of PCLV along with CFAV has implications for functional studies and in virus discovery, where Aag2 viruses could

contaminate environmental samples. Our findings emphasise the need for new mosquito cell lines that lack viral contaminants.

Detailed Materials and Methods for Virus Discovery

The 'non-insect' PIT data subset was searched against the GenBank database of viruses [taxid: 10239] using the online version of BLASTp (blast.ncbi.nlm.nih.gov). Only results with a good score (>50) were included; viral proteins with homology to TEs and cellular proteins were excluded. RNA-seq reads that did not map to the *Ae. aegypti* genome were mapped against the published CFAV [GenBank: KJ741267.1] and PCLV [GenBank: KM001085.1, KM001086.1, KM001087.1] reference genomes using Bowtie2 (galaxy.org), visualised using the Integrative Genomics Viewer (broadinstitute.org), and edited stylistically for clarity in Adobe Illustrator. Viral consensus sequences were constructed by analysis of the BAM files with Quasirecomb [14] to generate a phred-weighted table of nucleotide frequencies which were parsed with a custom Perl script to generate consensus sequences. Gaps were manually filled using RT-PCR amplicon sequences (>3X coverage). Sanger sequencing was performed by Macrogen USA (Rockville, MD USA). Homology to viral reference sequences was determined using a BLAST search against the CFAV [GenBank: KJ741267.1, AIM49245.1] and PCLV [GenBank: KM001085.1, KR003786.1, KM001086.1, KR003784.1, KM001087.1, KR003785.1, AIF71030.1, AKP18602.1, AIF71031.1, AKP18600.1, AIF71032.1, AKP18601.1] nucleotide and protein reference sequences. The CFAV and PCLV sequences determined in this study have been deposited in GenBank with the accession numbers KU936054 and KU936055, KU936056 and KU936057 respectively.

PCLV genome segments were amplified using the Maxima H Minus First Strand cDNA synthesis kit and AccuPrime Taq High Fidelity PCR kit (both ThermoFisher Scientific), as per manufacturer's instructions. PCR annealing temperatures and extension times were as follows; L, 58˚C, 7 min; M, 60˚C, 5 min; S, 60˚C, 1.5 min. Primers were as follows; L, prKM228F, ACACAAAGACGACGCATAAAATAAGC, prKM228R, ACCGACGCATGTTAATAAATGCTAAAC; M, prKM229F, ACACAAAGACAGCCCCTTAAGTAA, prKM229R, ACCAGCCCCACCATTGGAAAA; S, prKM230F, TGAAAGATTTGGTTACATTGTATTTTATAACT, prKM230R, CACAAAGACCAGCCCCAAATTTG.

ATC-10 cells were obtained from ATCC (Manassas, VA USA), ATC-15, C7/10 and RML-12 cells were kind gifts from Robert Tesh (University of Texas Medical Branch, Galveston, TX USA), *Ae. albopictus* C6/36 cells were a kind gift from Ana Fernandez-Sesma (Icahn School of Medicine at Mount Sinai, New York, NY USA) and *D. melanogaster* S2 cells were a kind gift from Raul Andino. Cells were maintained as for Aag2 cells (see main manuscript).

FIGURE S1

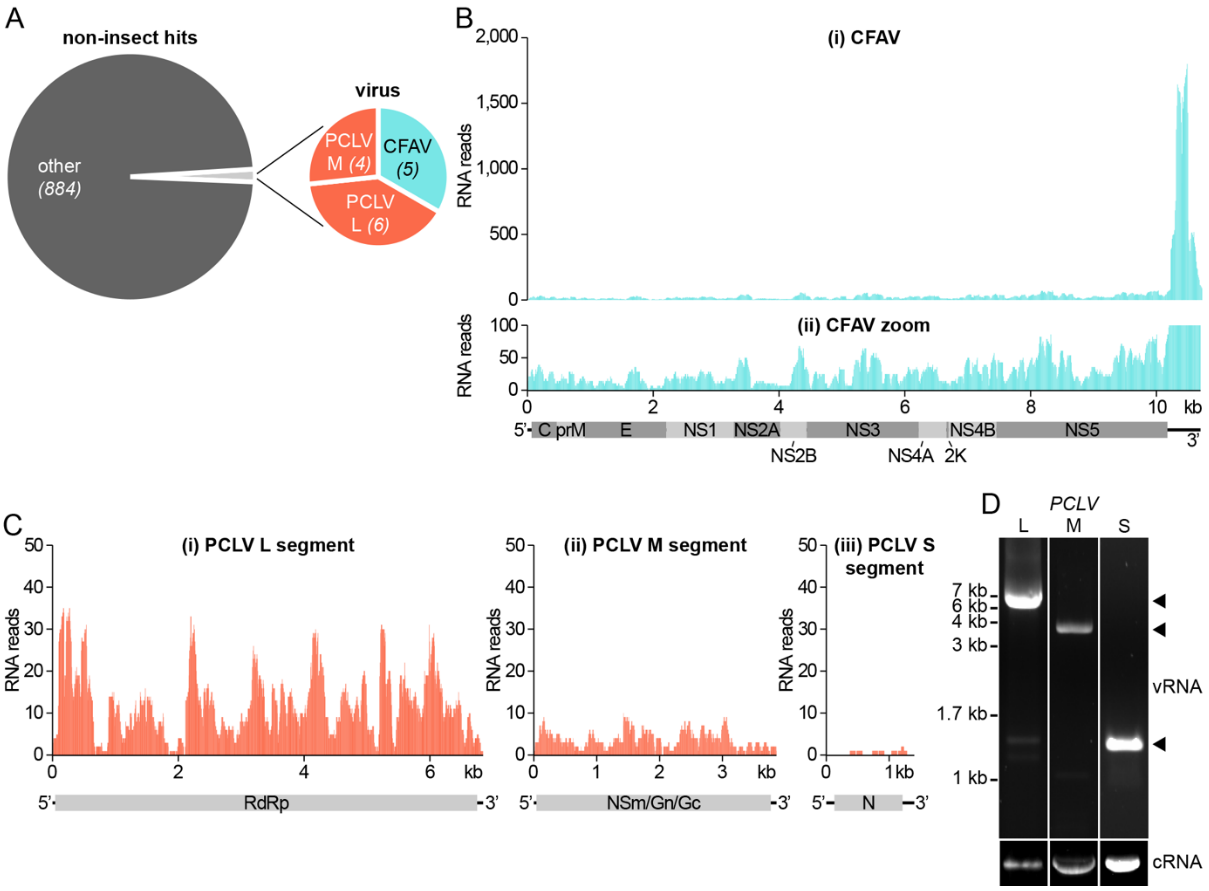

Fig. S1. Aag2 cells are persistently infected with two insect-specific viruses, CFAV and PCLV. (A) Detection of CFAV- and PCLV-derived proteins in the portion of the PIT proteome that does not map to insect genes (non-insect in Fig. 1Ci). (B and C) Distribution of RNA-seq reads across the CFAV genome (B) and PCLV large (L), medium (M) and small (S) genome segments (C). Approximate positions of viral genes and UTRs are shown for reference purposes. Viral sequences are shown in positive-sense; genome for CFAV, antigenome for PCLV. C, capsid; E, envelope; N, nucleocapsid; NS, non-structural protein; NSm/Gn/Gc, glycoprotein precursor; prM, pre-membrane; RdRp, RNA-dependent RNA polymerase. (D) PCR confirming the presence of all three full-length PCLV negative-sense viral genome (Vrna) and positive-sense complementary antigenome (Crna) segments in Aag2 cells.

REFERENCES

1. Stollar V, Thomas VL: **An agent in the Aedes aegypti cell line (Peleg) which causes fusion of Aedes albopictus cells.** *Virology* 1975, **64**:367–377.

2. Roby JA, Pijlman GP, Wilusz J, Khromykh AA: **Noncoding Subgenomic Flavivirus RNA: Multiple Functions in West Nile Virus Pathogenesis and Modulation of Host Responses.** *Viruses* 2014, **6**:404–427.

3. Cammisa-Parks H, Cisar LA, Kane A, Stollar V: **The complete nucleotide sequence of cell fusing agent (CFA): homology between the nonstructural proteins encoded by CFA and the nonstructural proteins encoded by arthropod-borne flaviviruses.** *Virology* 1992, **189**:511–524.

4. Walter CT, Barr JN: **Recent advances in the molecular and cellular biology of bunyaviruses.** *Journal of General Virology* 2011, **92**:2467–2484.

5. Hutchinson KL, Peters CJ, Nichol ST: **Sin Nombre virus mRNA synthesis.** *Virology* 1996, **224**:139–149.

6. Kolakofsky D, Hacker D: **Bunyavirus RNA synthesis: genome transcription and replication.** *Curr Top Microbiol Immunol* 1991, **169**:143–159.

7. Chandler JA, Thongsripong P, Green A, Kittayapong P, Wilcox BA, Schroth GP, Kapan DD, Bennett SN: **Metagenomic shotgun sequencing of a Bunyavirus in wild-caught Aedes aegypti from Thailand informs the evolutionary and genomic history of the Phleboviruses.** *Virology* 2014, **464-465C**:312–319.

8. Aguiar ERGR, Olmo RP, Paro S, Ferreira FV, de Faria IJDS, Todjro YMH, Lobo FP, Kroon EG, Meignin C, Gatherer D, Imler J-L, Marques JT: **Sequence-independent characterization of viruses based on the pattern of viral small RNAs produced by the host.** *Nucleic Acids Research* 2015, **43**:6191–6206.

9. Evans VC, Barker G, Heesom KJ, Fan J, Bessant C, Matthews DA: **De novo derivation of proteomes from transcriptomes for transcript and protein identification**. *Nature Methods* 2012, **9**:1–8.

10. Villar M, Popara M, Ayllón N, Fernández de Mera IG, Mateos-Hernández L, Galindo RC, Manrique M, Tobes R, la Fuente de J: **A systems biology approach to the characterization of stress response in Dermacentor reticulatus tick unfed larvae.** *PLoS One* 2014, **9**:e89564.

11. Sayama Y, Eshita Y, Yamao T, Nishimura M, Satho T, Srisawat R, Komalamisra N, Rongsriyam Y, Sakai K, Fukushi S, Saijo M, Oshitani H, Kurane I, Morikawa S, Mizutani T: **Prevalence of Phasi Charoen virus in female mosquitoes**. *Journal of Parasitology and Vector Biology* 2011, **3**:19–21.

12. Peleg J: **Growth of arboviruses in monolayers from subcultured mosquito embryo cells.** *Virology* 1968, **35**:617–619.

13. Lan Q, Fallon M: **Small heat shock proteins distinguish between two mosquito species and confirm identity of their cell lines**. *American Journal of Tropical Medicine and Hygiene* 1990, **43**:669–676.

14. Töpfer A, Zagordi O, Prabhakaran S, Roth V, Halperin E, Beerenwinkel N: **Probabilistic inference of viral quasispecies subject to recombination.** *J Comput Biol* 2013, **20**:113–123.
