# Supplementary material for: Proteomics informed by transcriptomics for characterising active transposable elements and genome annotation in Aedes aegypti
Source: BMC Genomics. 2017 Jan 19;18:101. doi: 10.1186/s12864-016-3432-5 (PMC5248466; doi:10.1186/s12864-016-3432-5)
Supplement: Additional file 6: — Published Proteomic and Transcriptomic Data Used in Analysis for Fig. 2B. (PDF 73 kb) [file 12864_2016_3432_MOESM6_ESM.pdf]

Published Proteomic and Transcriptomic Data Used in Analysis for Fig. 2B.

	Reference	Source	Sample	Technique
**proteomic**	[1]	table S1	larvae	LC-MS/MS
	[2]	table 1	adult +/- DENV	2D SDS-PAGE; MS[1]
	[3]	table S2	egg shells	LC-MS/MS
	[4]	tables 1 & 2	adult salivary gland	SDS-PAGE, MS[1]
	[5]	table 1	saliva	SDS-PAGE, MS[1]
	[6]	table 1	female & male reproductive tract	SDS-PAGE, MS[1]
	[7]	tables S1-S3, S5, S6	female reproductive tract, male sperm	LC-MS/MS
	[8]	table S2	adult midgut +/- CHIKV/DENV	2D SDS-PAGE; MS[1]
	[9]	tables S1-S4	adult salivary gland +/- CHIKV	2D SDS-PAGE; MS[1]
	[10]	supp. file 5	larval midgut; *Bti* resistant vs. susceptible	2D SDS-PAGE; MS[1]
	[11]	table S1	adult midgut +/- aa feed	LC-MS/MS
**transcriptomic**	[12]	table S3	different developmental stages	RNA-Seq
	[13]	table 6	adult; deltamethrin resistant vs. susceptible	microarray
	[14]	table S1	adult +/- blood meal	RNA-Seq
	[15]	tables S2 & S6	adult; different strains	RNA-Seq
	[16]	table S1	adult +/- DENV	RNA-Seq
	[17]	table S2	adult +/- blood meal	RNA-Seq
	[18]	tables S1 & S2	adult +/- *Wolbachia*	microarray
	[19]	datasheet S1	adult +/- *Brugia malayi*	RNA-Seq
	[20]	dataset S2	adult haemocytes vs. carcass; +/- bacteria	microarray
	[21]	table S1	adult +/- DENV, +/- WNV, +/- YFV	microarray
	[22]	table S2	larvae; pollutants & pesticides	RNA-Seq
	[23]	tables S3 & S5	adult +/- *B. malayi*	microarray
	[24]	table S2	adult	RNA-Seq
	[25]	table S3	larvae; temephos resistant vs. susceptible	microarray
	[26]	additional files 3 & 4	different developmental stages	microarray
	[27]	table S6	adults	RNA-Seq
	[28]	table S2	adult; *B. malayi* resistant vs. susceptible	RNA-Seq
	[29]	table 5	adult & larvae; pyrethroid resistant vs. susceptible	microarray
	[30]	tables S3 & S4	larvae; Cry11Aa resistant vs. susceptible	RNA-Seq
	[31]	supp. database	adult female	microarray
	[32]	supp. data	adult salivary gland +/- DENV	RNA-Seq
	[33]	table S1	adult & larvae; insecticide resistant vs. susceptible	microarray
	[3]	table S3	egg shells	RNA-Seq
	[34]	table S7	different developmental stages	MPSS
	[35]	tables S1-S4	adult female +/- *Wolbachia*	microarray
	[36]	table S2	larvae +/- xenobiotics	microarray
	[37]	tables S1 & S2	larvae; permethrin resistant vs. susceptible	microarray
	[38]	supp. file 1	fat body; small vs. large adults	RNA-Seq
	[39]	tables S2 & S3	adult female +/- *Wolbachia*	microarray
	[40]	appendix A	larvae +/- xenobiotics	microarray
	[41]	table S1	adult salivary gland	EST
	[42]	datasets S1 & S2, table S1	post-blood-meal fat body (adult female)	microarray
	[43]	tables 3-6	larvae; temephos resistant vs. susceptible	microarray
	[44]	tables S1 & S2	*Ae. aegypti* Aag2 cell line +/- DENV	microarray
	[45]	table S2	adult midgut & carcass	microarray
	[46]	tables S1-S4	adult salivary gland +/- DENV	microarray
	[47]	table S1	adult	microarray
	[10]	supp. file 1	larval midgut; *Bti* resistant vs. susceptible	microarray
	[48]	tables 1, S1, S2, S6	adult +/- DENV	microarray
	[49]	tables S1-S3, S5, S6	adult midgut & fat body +/- DENV, +/- *Plasmodium*	microarray
	[50]	table S1	adult fat body	microarray

[1] A subset of visible spots was picked for mass spectrometry analysis

aa, amino acid; *Bti*, *Bacillus thuringiensis* var. israelensis; CHIKV, chikungunya virus; DENV, dengue virus; EST, expressed sequence tag analysis; MPSS, massively parallel signature sequencing; supp., supplemental; WNV, West Nile virus; YFV, yellow fever virus.

REFERENCES

1. Bayyareddy K, Zhu X, Orlando R, Adang MJ: **Proteome analysis of Cry4Ba toxin-interacting Aedes aegypti lipid rafts using geLC-MS/MS.** *J Proteome Res* 2012, **11**:5843–5855.

2. Chisenhall DM, Londono BL, Christofferson RC, McCracken MK, Mores CN: **Effect of Dengue-2 Virus Infection on Protein Expression in the Salivary Glands of Aedes aegypti Mosquitoes.** *Am J Trop Med Hyg* 2014, **90**:431–437.

3. Marinotti O, Ngo T, Kojin BB, Chou S-P, Nguyen B, Juhn J, Carballar-Lejarazú R, Marinotti PN, Jiang X, Walter MF, Tu Z, Gershon PD, James AA: **Integrated proteomic and transcriptomic analysis of the Aedes aegypti eggshell.** *BMC Dev Biol* 2014, **14**:15.

4. Oktarianti R, Senjarini K, Hayano T, Fatchiyah F, Aulanni'am: **Proteomic analysis of immunogenic proteins from salivary glands of Aedes aegypti.** *J Infect Public Health* 2015.

5. Orlandi-Pradines E, Almeras L, Denis de Senneville L, Barbe S, Remoué F, Villard C, Cornelie S, Penhoat K, Pascual A, Bourgouin C, Fontenille D, Bonnet J, Corre-Catelin N, Reiter P, Pagés F, Laffite D, Boulanger D, Simondon F, Pradines B, Fusai T, Rogier C: **Antibody response against saliva antigens of Anopheles gambiae and Aedes aegypti in travellers in tropical Africa.** *Microbes Infect* 2007, **9**:1454–1462.

6. Sirot LK, Poulson RL, McKenna MC, Girnary H, Wolfner MF, Harrington LC: **Identity and transfer of male reproductive gland proteins of the dengue vector mosquito, Aedes aegypti: potential tools for control of female feeding and reproduction.** *Insect Biochemistry and Molecular Biology* 2008, **38**:176–189.

7. Sirot LK, Hardstone MC, Helinski MEH, Ribeiro JMC, Kimura M, Deewatthanawong P, Wolfner MF, Harrington LC: **Towards a semen proteome of the dengue vector mosquito: protein identification and potential functions.** *PLoS Negl Trop Dis* 2011, **5**:e989.

8. Tchankouo-Nguetcheu S, Khun H, Pincet L, Roux P, Bahut M, Huerre M, Guette C, Choumet V: **Differential protein modulation in midguts of Aedes aegypti infected with chikungunya and dengue 2 viruses.** *PLoS One* 2010, **5**:e13149.

9. Tchankouo-Nguetcheu S, Bourguet E, Lenormand P, Rousselle J-C, Namane A, Choumet V: **Infection by chikungunya virus modulates the expression of several proteins in Aedes aegypti salivary glands.** *Parasites & Vectors* 2012, **5**:264.

10. Tetreau G, Bayyareddy K, Jones CM, Stalinski R, Riaz MA, Paris M, David J-P, Adang MJ, Després L: **Larval midgut modifications associated with Bti resistance in the yellow fever mosquito using proteomic and transcriptomic approaches.** *BMC Genomics* 2012, **13**:248.

11. Zhou G, Isoe J, Day WA, Miesfeld RL: **Alpha-COPI coatomer protein is required for rough endoplasmic reticulum whorl formation in mosquito midgut epithelial cells.** *PLoS One* 2011, **6**:e18150.

12. Akbari OS, Antoshechkin I, Amrhein H, Williams B, Diloreto R, Sandler J, Hay BA: **The**

developmental transcriptome of the mosquito Aedes aegypti, an invasive species and major arbovirus vector. *G3 (Bethesda)* 2013, **3**:1493–1509.

13. Bingham G, Strode C, Tran L, Khoa PT, Jamet HP: **Can piperonyl butoxide enhance the efficacy of pyrethroids against pyrethroid-resistant Aedes aegypti?** *Trop Med Int Health* 2011, **16**:492–500.

14. Bonizzoni M, Dunn WA, Campbell CL, Olson KE, Dimon MT, Marinotti O, James AA: **RNA-seq analyses of blood-induced changes in gene expression in the mosquito vector species, Aedes aegypti.** *BMC Genomics* 2011, **12**:82.

15. Bonizzoni M, Britton M, Marinotti O, Dunn WA, Fass J, James AA: **Probing functional polymorphisms in the dengue vector, Aedes aegypti.** *BMC Genomics* 2013, **14**:739.

16. Bonizzoni M, Dunn WA, Campbell CL, Olson KE, Marinotti O, James AA: **Complex Modulation of the Aedes aegypti Transcriptome in Response to Dengue Virus Infection**. *PLoS One* 2012, **7**:e50512.

17. Bonizzoni M, Dunn WA, Campbell CL, Olson KE, Marinotti O, James AA: **Strain Variation in the Transcriptome of the Dengue Fever Vector, Aedes aegypti**. *G3* 2012, **2**:103–114.

18. Caragata EP, Poinsignon A, Moreira LA, Johnson PH, Leong YS, Ritchie SA, O'Neill SL, McGraw EA: **Improved accuracy of the transcriptional profiling method of age grading in Aedes aegypti mosquitoes under laboratory and semi-field cage conditions and in the presence of Wolbachia infection.** *Insect Molecular Biology* 2011, **20**:215–224.

19. Choi Y-J, Aliota MT, Mayhew GF, Erickson SM, Christensen BM: **Dual RNA-seq of parasite and host reveals gene expression dynamics during filarial worm-mosquito interactions.** *PLoS Negl Trop Dis* 2014, **8**:e2905.

20. Choi Y-J, Fuchs JF, Mayhew GF, Yu HE, Christensen BM: **Tissue-enriched expression profiles in Aedes aegypti identify hemocyte-specific transcriptome responses to infection.** *Insect Biochemistry and Molecular Biology* 2012, **42**:729–738.

21. Colpitts TM, Cox J, Vanlandingham DL, Feitosa FM, Cheng G, Kurscheid S, Wang P, Krishnan MN, Higgs S, Fikrig E: **Alterations in the Aedes aegypti Transcriptome during Infection with West Nile, Dengue and Yellow Fever Viruses**. *PLoS Pathog* 2011, **7**:e1002189.

22. David J-P, Coissac E, Melodelima C, Poupardin R, Riaz MA, Chandor-Proust A, Reynaud S: **Transcriptome response to pollutants and insecticides in the dengue vector Aedes aegypti using next-generation sequencing technology.** *BMC Genomics* 2010, **11**:216.

23. Erickson SM, Xi Z, Mayhew GF, Ramirez JL, Aliota MT, Christensen BM, Dimopoulos G: **Mosquito infection responses to developing filarial worms.** *PLoS Negl Trop Dis* 2009, **3**:e529.

24. Gibbons JG, Janson EM, Hittinger CT, Johnston M, Abbot P, Rokas A: **Benchmarking next-generation transcriptome sequencing for functional and evolutionary genomics.** *Mol Biol Evol* 2009, **26**:2731–2744.

25. Grisales N, Poupardin R, Gomez S, Fonseca-Gonzalez I, Ranson H, Lenhart A: **Temephos resistance in Aedes aegypti in Colombia compromises dengue vector control.** *PLoS Negl Trop Dis* 2013, **7**:e2438.

26. Harker BW, Behura SK, Debruyn BS, Lovin DD, Mori A, Romero-Severson J, Severson

DW: **Stage-specific transcription during development of Aedes aegypti.** *BMC Dev Biol* 2013, **13**:29.

27. Jiang X, Biedler JK, Qi Y, Hall AB, Tu Z: **Complete Dosage Compensation in Anopheles stephensi and the Evolution of Sex-Biased Genes in Mosquitoes.** *Genome Biol Evol* 2015, **7**:1914–1924.

28. Juneja P, Ariani CV, Ho YS, Akorli J, Palmer WJ, Pain A, Jiggins FM: **Exome and Transcriptome Sequencing of Aedes aegypti Identifies a Locus That Confers Resistance to Brugia malayi and Alters the Immune Response.** *PLoS Pathog* 2015, **11**:e1004765.

29. Kasai S, Komagata O, Itokawa K, Shono T, Ng LC, Kobayashi M, Tomita T: **Mechanisms of pyrethroid resistance in the dengue mosquito vector, Aedes aegypti: target site insensitivity, penetration, and metabolism.** *PLoS Negl Trop Dis* 2014, **8**:e2948.

30. Lee S-B, Aimanova KG, Gill SS: **Alkaline phosphatases and aminopeptidases are altered in a Cry11Aa resistant strain of Aedes aegypti.** *Insect Biochemistry and Molecular Biology* 2014, **54**:112–121.

31. Leming MT, Rund SSC, Behura SK, Duffield GE, O'Tousa JE: **A database of circadian and diel rhythmic gene expression in the yellow fever mosquito Aedes aegypti.** *BMC Genomics* 2014, **15**:1128.

32. Luplertlop N, Surasombatpattana P, Patramool S, Dumas E, Wasinpiyamongkol L, Saune L, Hamel R, Bernard E, Sereno D, Thomas F, Piquemal D, Yssel H, Briant L, Missé D: **Induction of a Peptide with Activity against a Broad Spectrum of Pathogens in the Aedes aegypti Salivary Gland, following Infection with Dengue Virus**. *PLoS Pathog* 2011, **7**:e1001252.

33. Marcombe S, Poupardin R, Darriet F, Reynaud S, Bonnet J, Strode C, Brengues C, Yébakima A, Ranson H, Corbel V, David J-P: **Exploring the molecular basis of insecticide resistance in the dengue vector Aedes aegypti: a case study in Martinique Island (French West Indies).** *BMC Genomics* 2009, **10**:494.

34. Nene V, Wortman JR, Lawson D, Haas B, Kodira C, Tu ZJ, Loftus B, Xi Z, Megy K, Grabherr M, Ren Q, Zdobnov EM, Lobo NF, Campbell KS, Brown SE, Bonaldo MF, Zhu J, Sinkins SP, Hogenkamp DG, Amedeo P, Arensburger P, Atkinson PW, Bidwell S, Biedler J, Birney E, Bruggner RV, Costas J, Coy MR, Crabtree J, Crawford M, et al.: **Genome sequence of Aedes aegypti, a major arbovirus vector.** *Science Reports* 2007, **316**:1718–1723.

35. Pan X, Zhou G, Wu J, Bian G, Lu P, Raikhei AS, Xi Z: **Wolbachia induces reactive oxygen species (ROS)-dependent activation of the Toll pathway to control dengue virus in the mosquito Aedes aegypti**. *Proceedings of the National Academy of Sciences* 2011:1–9.

36. Poupardin R, Reynaud S, Strode C, Ranson H, Vontas J, David J-P: **Cross-induction of detoxification genes by environmental xenobiotics and insecticides in the mosquito Aedes aegypti: impact on larval tolerance to chemical insecticides.** *Insect Biochemistry and Molecular Biology* 2008, **38**:540–551.

37. Poupardin R, Riaz MA, Jones CM, Chandor-Proust A, Reynaud S, David J-P: **Do pollutants affect insecticide-driven gene selection in mosquitoes? Experimental evidence from transcriptomics.** *Aquat Toxicol* 2012, **114-115**:49–57.

38. Price DP, Schilkey FD, Ulanov A, Hansen IA: **Small mosquitoes, large implications: crowding and starvation affects gene expression and nutrient accumulation in Aedes**

aegypti. *Parasites & Vectors* 2015, **8**:252.

39. Rancès E, Ye YH, Woolfit M, McGraw EA, O'Neill SL: **The relative importance of innate immune priming in Wolbachia-mediated dengue interference.** *PLoS Pathog* 2012, **8**:e1002548.

40. Riaz MA, Poupardin R, Reynaud S, Strode C, Ranson H, David J-P: **Impact of glyphosate and benzo[a]pyrene on the tolerance of mosquito larvae to chemical insecticides. Role of detoxification genes in response to xenobiotics.** *Aquat Toxicol* 2009, **93**:61–69.

41. Ribeiro JMC, Arcà B, Lombardo F, Calvo E, Phan VM, Chandra PK, Wikel SK: **An annotated catalogue of salivary gland transcripts in the adult female mosquito, Aedes aegypti.** *BMC Genomics* 2007, **8**:6.

42. Roy S, Saha TT, Johnson L, Zhao B, Ha J, White KP, Girke T, Zou Z, Raikhel AS: **Regulation of Gene Expression Patterns in Mosquito Reproduction.** *PLoS Genet* 2015, **11**:e1005450.

43. Saavedra-Rodriguez K, Strode C, Flores AE, Garcia-Luna S, Reyes-Solis G, Ranson H, Hemingway J, Black WC: **Differential transcription profiles in Aedes aegypti detoxification genes after temephos selection.** *Insect Molecular Biology* 2014, **23**:199–215.

44. Sim S, Dimopoulos G: **Dengue Virus Inhibits Immune Responses in Aedes aegypti Cells**. *PLoS One* 2010, **5**:e10678.

45. Sim S, Jupatanakul N, Ramirez JL, Kang S, Romero-Vivas CM, Mohammed H, Dimopoulos G: **Transcriptomic Profiling of Diverse Aedes aegypti Strains Reveals Increased Basal-level Immune Activation in Dengue Virus-refractory Populations and Identifies Novel Virus-vector Molecular Interactions.** *PLoS Negl Trop Dis* 2013, **7**:e2295.

46. Sim S, Ramirez JL, Dimopoulos G: **Dengue Virus Infection of the Aedes aegypti Salivary Gland and Chemosensory Apparatus Induces Genes that Modulate Infection and Blood-Feeding Behavior**. *PLoS Pathog* 2012, **8**:e1002631.

47. Souza-Neto JA, Sim S, Dimopoulos G: **An evolutionary conserved function of the JAK-STAT pathway in anti-dengue defense**. *Proceedings of the National Academy of Sciences* 2009, **106**:17841–17846.

48. Xi Z, Ramirez JL, Dimopoulos G: **The Aedes aegypti toll pathway controls dengue virus infection.** *PLoS Pathog* 2008, **4**:e1000098.

49. Zou Z, Souza-Neto J, Xi Z, Kokoza V, Shin SW, Dimopoulos G, Raikhel A: **Transcriptome analysis of Aedes aegypti transgenic mosquitoes with altered immunity.** *PLoS Pathog* 2011, **7**:e1002394.

50. Zou Z, Saha TT, Roy S, Shin SW, Backman TWH, Girke T, White KP, Raikhel AS: **Juvenile hormone and its receptor, methoprene-tolerant, control the dynamics of mosquito gene expression.** *Proceedings of the National Academy of Sciences* 2013, **110**:E2173–81.
