# Supplementary material for: Proteomics informed by transcriptomics for characterising active transposable elements and genome annotation in Aedes aegypti
Source: BMC Genomics. 2017 Jan 19;18:101. doi: 10.1186/s12864-016-3432-5 (PMC5248466; doi:10.1186/s12864-016-3432-5)
Supplement: Additional file 7: — Ae. aegypti-Mapped PIT Hits Used in Analysis for Fig. 3A. (PDF 14 kb) [file 12864_2016_3432_MOESM7_ESM.pdf]

***Ae. aegypti*-Mapped PIT Hits Used in Analysis for Figure 3A.**

(Listed by Trinity ID).

93, 145, 154, 209, 259, 268, 288, 289, 346, 403, 548, 558, 576, 593, 632, 753, 788, 1000, 1011, 1012, 1019, 1034, 1095, 1138, 1214, 1224, 1256, 1284, 1385, 1392, 1697, 1931, 1983, 2094, 2114, 2118, 2203, 2286, 2328, 2371, 2380, 2461, 2498, 2580, 2588, 2593, 2699, 2775, 2843, 2850, 2855, 2954, 2999, 3039, 3196, 3232, 3268, 3354, 3444, 3450, 3478, 3518, 3614, 3883, 3926, 4023, 4073, 4104, 4119, 4234, 4253, 4276, 4314, 4380, 4647, 4678, 4730, 4806, 4951, 4952, 4955, 5015, 5129, 5130, 5211, 5256, 5399, 5456, 5517, 5562, 5577, 5627, 5738, 5803, 5865, 6073, 6077, 6232, 6303, 6488, 6628, 6706, 6731, 6732, 6735, 6760, 6917, 7250, 7334, 7348, 7391, 7396, 7531, 7616, 7631, 7654, 7673, 7732, 7812, 7860, 7865, 7891, 7960, 7966, 7975, 7997, 8008, 8137, 8139, 8164, 8279, 8438, 8607, 8619, 8622, 8810, 8949, 8961, 8996, 9090, 9113, 9151, 9221, 9316, 9369, 9400
